# Comparative Analysis Among Different Species Reveals That the Androgen Receptor Regulates Chicken Follicle Selection Through Species-Specific Genes Related to Follicle Development

**DOI:** 10.3389/fgene.2021.752976

**Published:** 2022-01-03

**Authors:** Ying Huang, Wei Luo, Xuliang Luo, Xiaohui Wu, Jinqiu Li, Yan Sun, Shuixin Tang, Jianhua Cao, Yanzhang Gong

**Affiliations:** ^1^ Key Laboratory of Agricultural Animal Genetics, Breeding and Reproduction (Huazhong Agricultural University), Ministry of Education, Wuhan, China; ^2^ Guilin Medical University, Guilin, China; ^3^ Central Laboratory, Affiliated Hospital of Putian University, Putian, China

**Keywords:** chicken, androgen receptor, follicle development, transcriptome, super-enhancer

## Abstract

The differences in reproductive processes at the molecular level between viviparous and oviparous animals are evident, and the site in the ovary that synthesizes sex hormones (androgens and oestrogens) and the trends for enriching sex hormones during follicle development in chickens are different from those in mammals, suggesting that the effect of sex hormones on follicle development in chickens is probably different from that in viviparous animals. To explore the specific role of androgen receptors (ARs) on chicken follicular development, we matched the correspondence of follicular development stages among chickens, humans, cows and identified chicken-specific genes related to follicle development (GAL-SPGs) by comparing follicle development-related genes and their biological functions among species (chickens, humans, and cows). A comparison of the core transcription factor regulatory network of granulosa cells (or ovaries) based on super-enhancers among species (chicken, human, and mouse) revealed that AR is a core transcriptional regulator specific to chickens. *In vivo* experiments showed that inhibition of AR significantly reduced the number of syf (selected stage follicles) in chickens and decreased the expression of GAL-SPGs in F5 follicles, while *in vitro* experiments showed that inhibition of AR expression in chicken granulosa cells (GCs) significantly down-regulated the expression levels of GAL-SPGs, indicating that AR could regulate follicle selection through chicken-specific genes related to follicle development. A comparison among species (77 vertebrates) of the conserved genomic regions, where chicken super-enhancers are located, revealed that the chicken AR super-enhancer region is conserved in birds, suggesting that the role of AR in follicle selection maybe widespread in birds. In summary, we found that AR can regulate follicle selection through chicken-specific genes related to follicle development, which also emphasizes the important role of AR in follicle selection in chickens and provides a new perspective for understanding the unique process of follicle development in chickens. Our study will contribute to the application of androgens to the control of egg production in chickens and suggests that researchers can delve into the mechanisms of follicle development in birds based on androgen/androgen receptors.

## Introduction

Differences in reproductive processes at the molecular level between viviparous and oviparous animals are evident, and follicular development is both the starting point and the basis for subsequent reproductive processes. The follicles of mammals, represented by humans, are divided into five stages based on their histomorphology and diameter, i.e., primordial, primary, secondary, antral, and preovulatory follicles (Gougeon, 1986). However, the follicles in chickens are classified according to their sizes, i.e., small white follicles (swf), large white follicles (lwf), small yellow follicles (syf), and preovulatory follicles (po). The syfs are also known as pre-hierarchical follicles (ph), and the preovulatory follicles can be marked as F1- F5 follicles in descending order of diameter (Johnson, 2015). The follicular development stages in chickens do not fully correspond to the stages of mammalian follicular development. Follicle selection is an important event during follicular development that determines whether the follicle can enter the preovulatory stage and continue to develop to ovulation. Antral follicles of humans at 2–10 mm (Ginther et al., 2001) and small yolk follicles of chickens at 6–8 mm (Johnson, 2012) are considered to be in the follicular selection stage. The typical structure of follicles is that a developing oocyte is surrounded by follicular somatic cells, which include the granulosa cell layer and thecal cell layers. Generally, granulosa cells are believed to play important roles in follicle selection.

Previous studies have shown that enhancers are more related to phenotype than other DNA regulatory elements (Yue et al., 2014). Super-enhancers control potentially key cell identity genes that maintain cell identity and cell states. The molecular function of genes associated with super-enhancers is primarily transcriptional regulation, in which they can form a set of transcriptional regulatory circuits, and the transcription factors at the nodes of the circuits are probably the key factors for maintaining the cellular state (Hnisz et al., 2013; Saint-André et al., 2016; Whyte et al., 2013). Therefore, a core transcription factor regulatory network constructed from granulosa cell (GC) super-enhancers facilitates the identification of genes that are essential for follicle development. Sex hormones, including oestrogens and androgens, play a vital role in follicular development (Condon et al., 2020; Çiftci, 2017; Couse et al., 2005; Hu et al., 2004; Shiina et al., 2006; Sen et al., 2014; Tanaka et al., 2017). However, the sex hormones in chickens differ from those in mammals due to their synthesis sites and enrichment trends. The main manifestations are as follows: (i) oestrogens and their receptors synthesized in the follicular granulosa cells (GCs) of human follicles (Hillier et al., 1994) are synthesized in the theca cells (TCs) of chicken follicles (Bahr et al., 1983; Gan et al., 2017; Wang and Gong, 2017), and (ii) opposite trends of sex hormone enrichments in the preovulatory follicular stage between humans and chickens are presented (Kristensen et al., 2017; Li et al., 2019). During follicular development, the follicular fluid of humans has progressively higher levels of oestrogens and lower levels of androgens, whereas the follicular fluid of chickens has progressively lower levels of oestrogens and higher levels of androgens (Supplementary Figure S1). This suggests that androgen/androgen receptor (AR) signalling in chickens might differ from that in mammals. In mammals, knockdown of androgens significantly inhibits folliculogenesis (Shiina et al., 2006), whereas excess androgens lead to polycystic follicle syndrome (PCOS) via extra ovarian mediators (Caldwell et al., 2017). To explore the specific role of AR in chicken follicular development, a set of chicken-specific genes related to follicle development was identified by comparing follicle development-related genes and their biological functions among species (chickens, humans, and cows). By comparing the core transcription factor regulatory network of GCs (or ovaries) based on super-enhancers among species (chickens, humans, and mice), AR was found to be a core transcriptional regulator specific to chickens. *In vivo* and *in vitro* experiments showed that inhibition of AR significantly down-regulated GAL-SPGs and reduced the number of chicken syf (selected stage follicles). Our findings highlight the important role of AR, which regulates follicle selection through chicken-specific genes related to follicle development and explain the phenomenon of differential follicle hormone levels between chickens and mammals.

## Results

### Inconsistent Correspondence of Preovulatory Follicles Among Chickens, Cows and Humans

**FIGURE 1:**
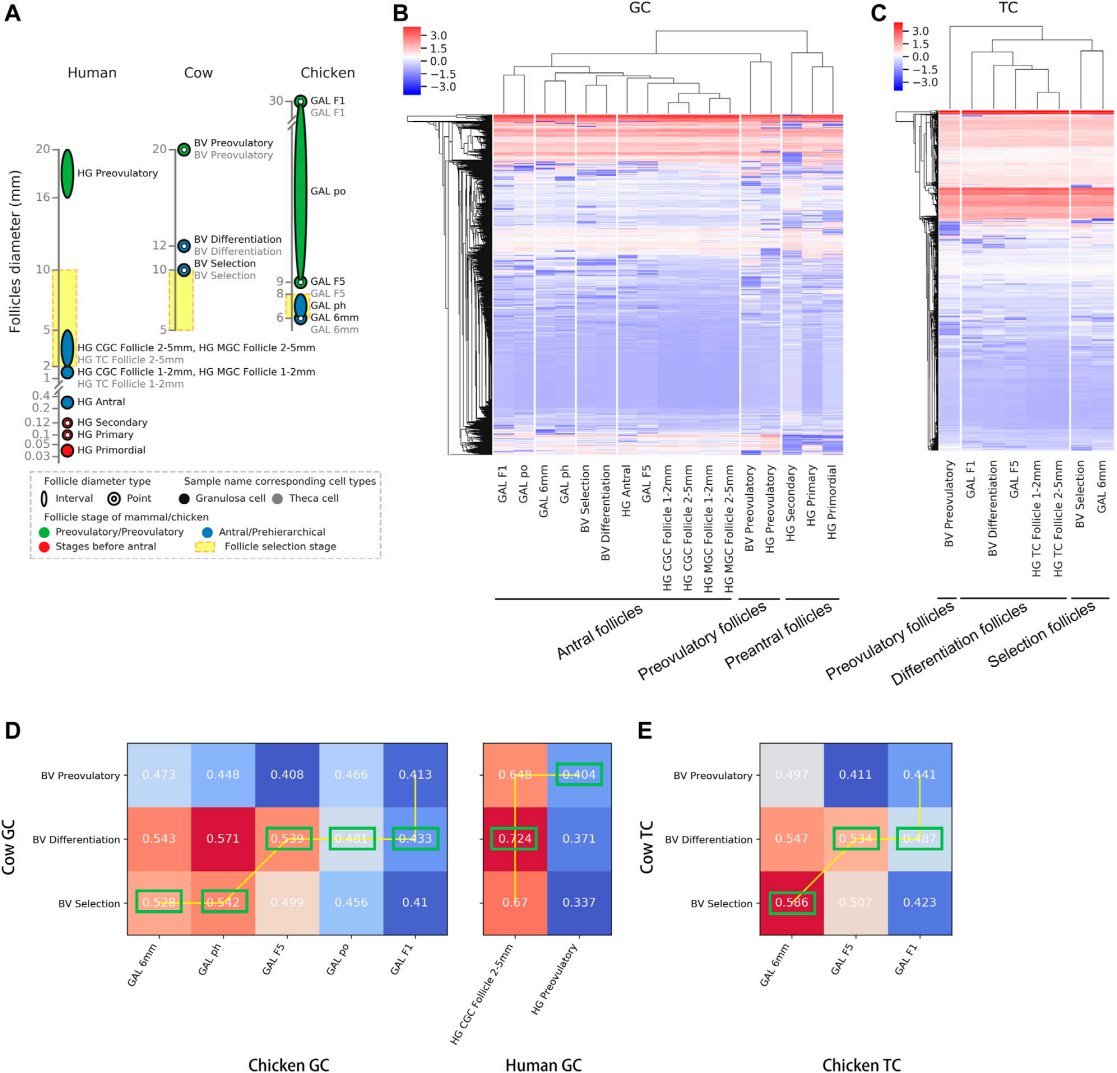
Correspondence between the stages of follicle development in oviparous and viviparous animals. **(A)** Species and stages of follicle samples. Each circle or ellipse represents a type of sample. **(B,C)** Hierarchical clustering of GCs and TCs based on gene expression at each stage of follicle development for each species. The colours in the heatmap correspond to gene expression after standardization of the z-score. **(D,E)** Correlations (Spearman’s correlation coefficients) of GCs and TCs at each developmental stage after follicle selection between chickens and bovines and between humans and bovines. The best alignments are connected by a yellow line, and the best corresponding stages are marked by green boxes.

To clarify the corresponding relationship of follicle development stages among chickens, cows and humans, we first compared the correspondence between the various stages of follicular development among species based on the transcriptomic data. The GC and TC transcriptomic data from the GEO database (human and bovine) (Walsh et al., 2012; Zhang et al., 2018; Fan et al., 2019) and our laboratory (chicken, Supplementary Table S1) were integrated (Figure 1A), and a total of 15,088 homologous genes (FPKM ≥ 1) were obtained (see Supplementary Table S2 for details). After removing the batch effect (Supplementary Figure S2A), hierarchical clustering was performed on the samples (shown at the top of Figures 1B,C), and a homologous gene expression matrix was displayed as a heatmap (shown at the bottom of Figures 1B,C; Supplementary Table S2). In the GCs, taking the human follicle samples as a reference, all samples were clustered into three groups and named “preantral follicles” (primordial, primary and secondary follicles), “antral follicles”, or “preovulatory follicles” based on the results of hierarchical clustering (Figure 1B). Among them, the antral follicles (BV selection and BV differentiation) and preovulatory follicles (BV preovulatory) of bovines were divided into an “antral follicle” cluster and a “preovulatory follicle” cluster, respectively, showing that the classification results were reasonable. In contrast, chicken preovulatory follicles (GAL F1, GAL po, and GAL F5) were classified into the “antral follicle” cluster. In the TCs, as human follicle samples cover fewer developmental stages, taking the bovine follicle samples as a reference, based on the results of hierarchical clustering, all samples were also clustered into three groups: “selection follicles”, “differentiation follicles” and “preovulatory follicles” (Figure 1C). The results showed that the pre-hierarchical follicles (GAL 6 mm) and preovulatory follicles (GAL F1 and GAL F5) of chickens were divided into a “selection follicle” cluster and a “differentiation follicle” cluster, respectively, which are known as the antral follicle stage in bovines. Interestingly, F5 follicles in the early stage of preovulatory follicles in chickens had the highest similarity to human antral follicles other than other stages of chicken follicles.

To further accurately match the order of developmental stages among species, we collected and compared the correspondence of common follicular developmental stages (the selection, differentiation, and preovulation stages) across species using dynamic time warping (DTW) (Figures 1D,E). Due to the lack of data on TCs in human preovulatory follicles, only the correspondence of common follicle development stages between chickens and bovines was compared in TCs. The follicular development stages in cattle were used as a reference. In GCs, the follicles best matched to the bovine selection follicles were chicken pre-hierarchical follicles (GAL 6 mm and GAL ph); the follicles best matched to the bovine differentiation follicles were chicken preovulatory follicles (GAL F5, GAL po, and GAL F1) and human antral follicles (HG CGC follicle 2–5 mm); the follicles best matched to the bovine preovulatory follicles were human preovulatory follicles (HG preovultory) (Figure 1D, marked with green box). In TCs, the follicles best matched to the bovine selection follicles were chicken pre-hierarchical follicles (GAL 6 mm); the follicles best matched to the bovine differentiation follicles were chicken preovulatory follicles (GAL F5 and GAL F1) (Figure 1E).

The above results showed that chicken pre-hierarchical follicles and preovulatory follicles both corresponded to mammalian antral follicles, suggesting immaturity of the chicken preovulatory follicles. Interestingly, F5 follicles in the early stage of preovulatory follicles in chickens had the highest similarity to human antral follicles rather than other stages of chicken follicles.

### Comparison of Genes Related to Follicle Development Among Chickens, Cows and Humans

**FIGURE 2:**
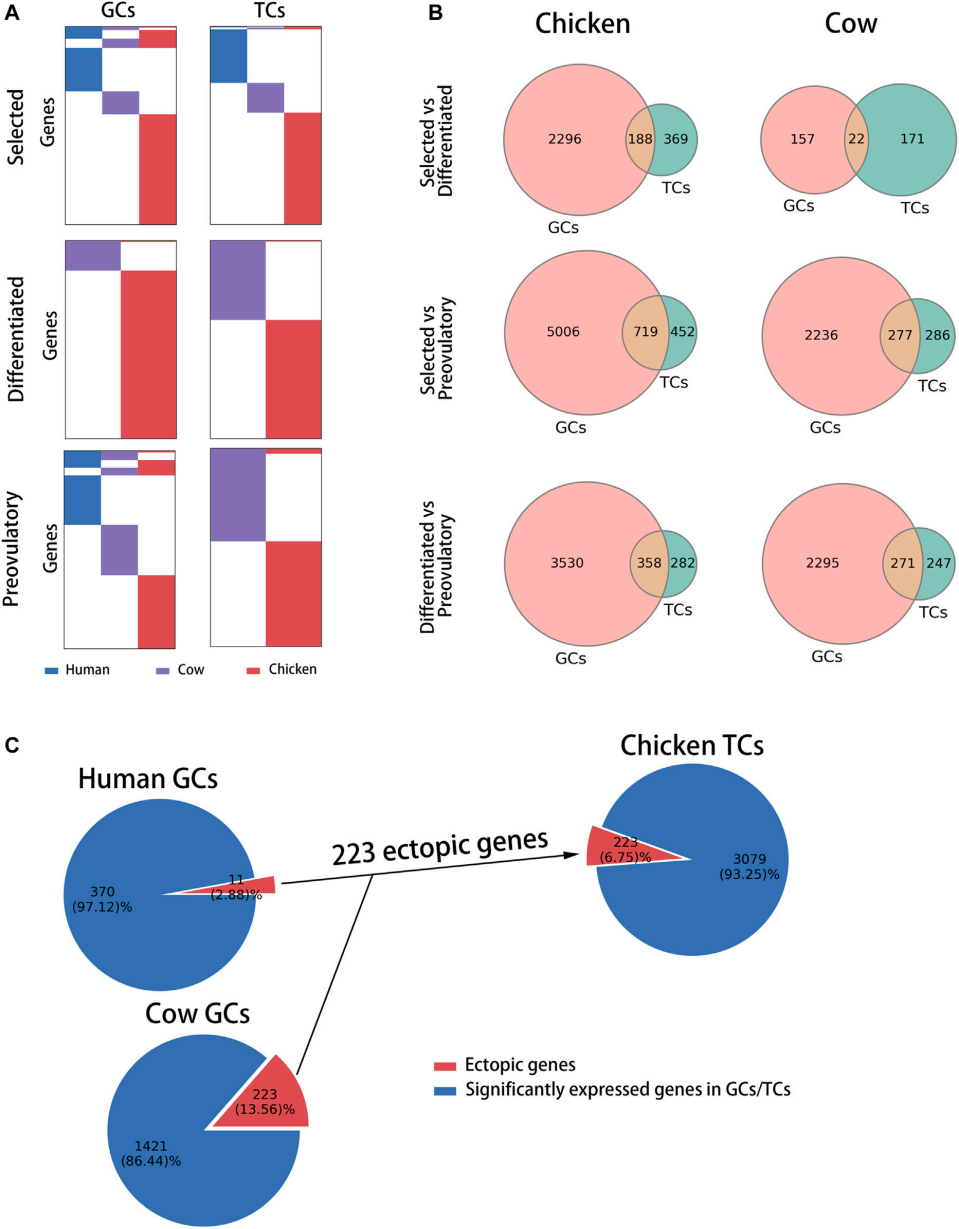
Comparison of follicle development-related genes among species at each stage of follicle development. **(A)** Distribution of follicle development-related genes (rows) among species (columns) at each stage in GCs and TCs. Human, cow and chicken genes are marked in blue, purple and red, respectively. **(B)** Venn diagram showing the distribution of follicle development-related genes between chickens and cows at each stage in GCs and TCs. **(C)** Pie charts showing the percentage of ectopically expressed genes (red) at each stage for each species.

To compare follicle development-related genes in chickens, cows and humans, the differentially highly expressed genes at each stage (selected stage, differentiated stage, and preovulatory stage) were identified as follicle development-related genes based on the transcriptomes of GCs and TCs at each stage in each species (see Supplementary Table S3 for details). A comparison of the homology of follicle development-related genes between species at each stage showed that, at the selected stage, 10.80 and 1.43% of follicle development-related genes were homologous in GCs and TCs, respectively (Figure 2A, top of the graphic); at the differentiated stage, 0.73 and 0.54% of follicle development-related genes were homologous in GCs and TCs, respectively (Figure 2A, middle of the graphic); and at the preovulatory stage, 12.49 and 0.69% of follicle development-related genes were homologous in GCs and TCs, respectively (Figure 2A, bottom of the graphic). These results suggest that follicle development-related genes are species-specific. A comparison of the distribution of follicle development-related genes in GCs and TCs between chickens and cows showed that 2,296, 5,006, and 3,530 genes in chickens (Figure 2B, left of the graphic, red circles) and 157, 2,236, and 2,295 genes in cows (Figure 2B, right of the graphic, red circles) were expressed in GCs only at each respective stage; meanwhile, 369, 452, and 282 genes in chickens (Figure 2B, left of the graphic, green circles) and 171, 286, and 247 genes in cows (Figure 2B, right of the graphic, green circles) were expressed in TCs only at each respective stage, suggesting that in general, follicle development-related genes are predominantly expressed in GCs. Previous studies have shown that the genes (CYP19A1 and ESR2) predominantly expressed in mammalian GCs are only expressed in chicken TCs (Wang and Gong, 2017; Jing et al., 2018), and we define them as ectopically expressed genes. A comparison of the distribution of these ectopically expressed genes among species revealed that 2.88, 13.56 and 6.75% of the human, cow and chicken follicle development-related genes were ectopically expressed (Figure 2C), respectively, indicating that a smaller proportion of ectopically expressed genes occurred among species.

### Identification of Follicle Development-Related Genes Specific to Chickens

**FIGURE 3:**
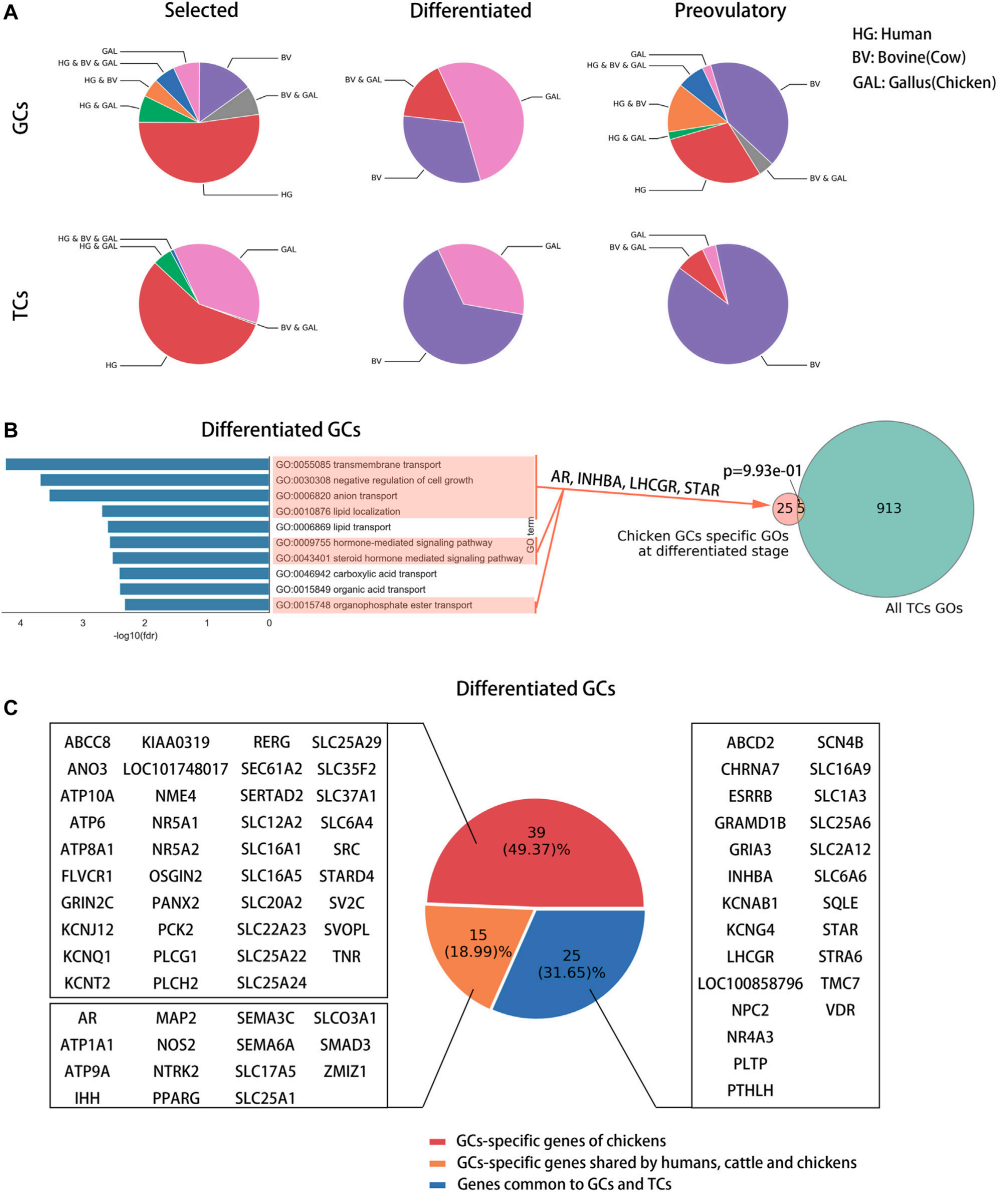
Identification of follicle development-related genes specific to chickens. **(A)** Pie charts showing the percentage of follicle development-related BPs at each stage for each species. **(B)** Candidate chicken-specific BPs in GCs at the differentiated stage. On the left of the graph are the top ten BPs specific to chickens in GCs at the differentiated stage, in which candidate chicken-specific BPs are marked in red. On the right of the graph are the Venn diagram showing the overlap between galBP-diffGCs (red) and all BPs in TCs (green). *p* was calculated using hypergeometric test, *p* ≤ 0.0001. Candidate chicken-specific BPs were found only in galBP-diffGCs, marked with red arrows, and these BPs were used to identify candidate chicken-specific follicle development-related genes. Previously reported follicle development-related genes are marked with red arrows. **(C)** Pie chart showing the percentage and corresponding gene names of candidate chicken-specific follicle development-related genes (red) identified from candidate chicken-specific BPs in galBP-diffGCs.

To identify follicle development-related genes specific to chickens, we compared the biological functions of follicle development-related genes at each stage across species (Supplementary Table S4) to exclude the possibility that different genes perform similar biological functions among species. Nineteen, 30 and 24 chicken-specific biological processes (BPs) were identified at the selected, differentiated and preovulatory stages of GCs, respectively; meanwhile, 132, 12 and 13 chicken-specific biological processes (BPs) were identified at the selected, differentiated and preovulatory stages of GCs, respectively (Supplementary Table S4). Biological processes specific to chickens were more predominant in GCs at the differentiated stage (galBPs-diffGCs, 52.41%), in TCs at the selected stage (galBPs-slctTCs, 37.09%), and in TCs at the differentiated stage (galBPs-diffTCs, 34.72%) compared with the other stages (Figure 3A), and they were used as candidate chicken-specific BPs. To exclude BPs present in both GCs and TCs, we compared the distribution of candidate chicken-specific BPs (galBPs) in GCs and TCs. The results showed that the galBPs-slctTCs significantly overlapped (*p* ≤ 0.0001) with BPs in the GCs (Supplementary Figure S3A, upper right of the graphic), whereas the galBPs-diffGCs and galBPs-diffTCs did not significantly (*p* = 0.9930 and *p* = 0.6900) overlap with BPs in the TCs or GCs (Figure 3B, right of the graphic; Supplementary Figure S3A, lower right of the graphic), indicating that galBPs-diffGCs and galBPs-diffTCs mainly existed in GCs and TCs, respectively. BPs present only in GCs from the top ten galBPs-diffGCs (ordered by the *p*-value of GO enrichment) (Figure 3B, left of the graphic) and BPs present only in TCs from the top ten galBPs-diffTCs (Supplementary Figure S3A, lower left of the graphic) were used to identify candidate chicken-specific follicle development-related genes. A total of 6.25% (1 gene) of the candidate genes identified from galBPs-diffTCs were found only in TCs (Supplementary Figure S3B, red part of pie), whereas 49.37% (39 genes) of the candidate genes identified from galBPs-diffGCs were found only in GCs (Figure 3C, red part of pie); thus, the genes found only in GCs from galBPs-diffGCs were considered chicken-specific follicle development-related genes (GAL-SPGs, Figure 3C, red part of pie). GAL-SPGs were significantly highly expressed genes (*p*adj ≤ 0.05) in GCs at the differentiated stage (F5) compared with the selected stage (syf 6 mm) (Supplementary Figure S4A). The gene set of GAL-SPGs was significantly up-regulated (*p* ≤ 0.01) in GCs with high FSHR expression (Supplementary Figure S4B, public data from (Wang et al., 2017)), and two GAL-SPGs were significantly highly expressed genes (*p*adj ≤ 0.05) in GCs with high FSHR expression compared with GCs with low FSHR expression (Supplementary Figure S4C), suggesting that GAL-SPGs maybe associated with follicular selection. The GO analysis revealed that GAL-SPGs were mainly enriched in transport-related biological processes (Supplementary Figure S4D).

### AR Is a Core Transcriptional Regulator Specific to Chickens in GCs

**FIGURE 4:**
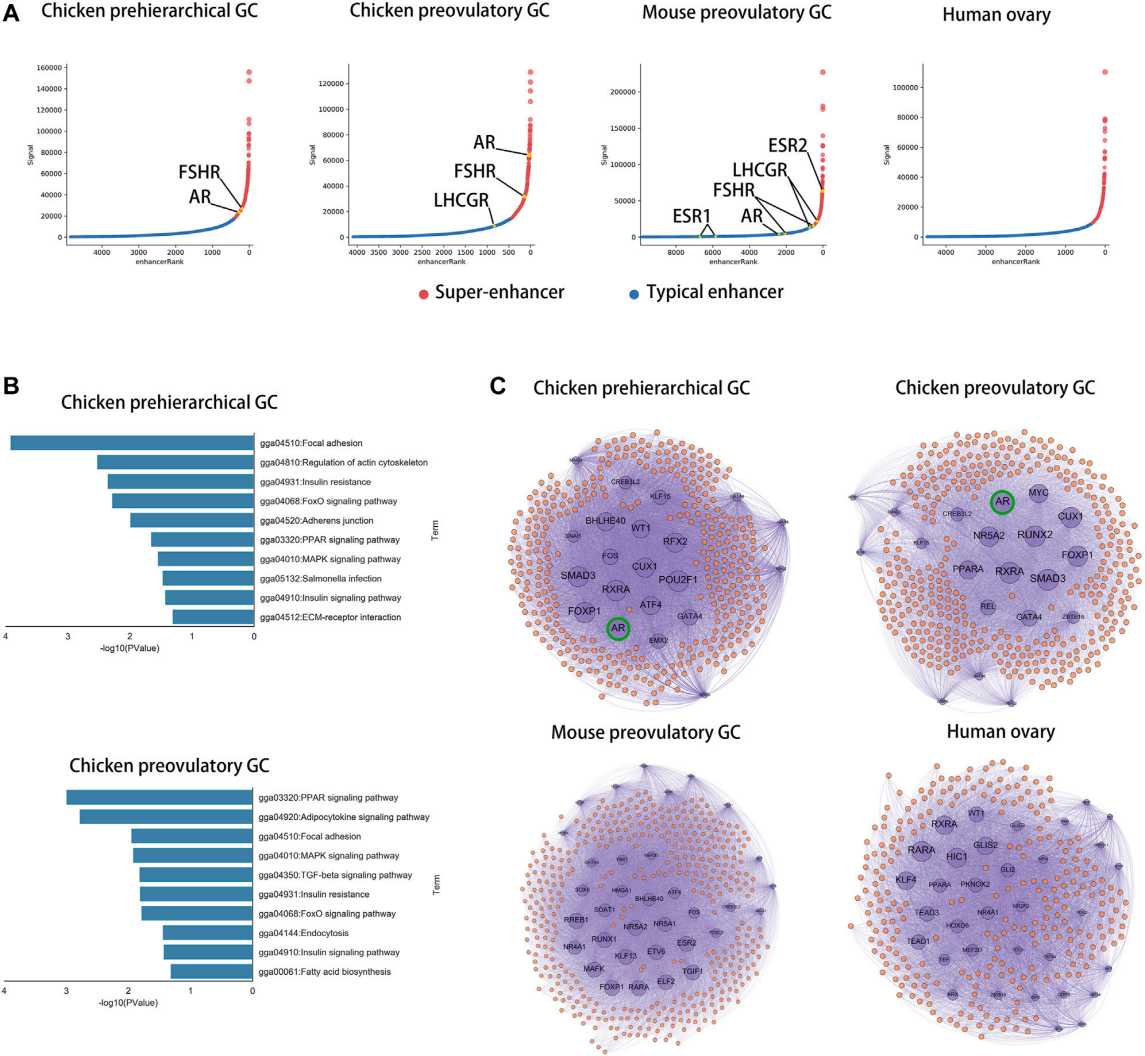
AR is a core transcriptional regulator specific to chickens in GCs. **(A)** Super-enhancers (red) and typical enhancers (blue) identified by ROSE in chicken phGCs, chicken poGCs, mouse poGCs and human ovaries. Enhancer-associated hormone receptor gene names are tagged (orange). **(B)** Top ten KEGG pathways of SEGs in chicken phGCs and chicken poGCs. **(C)** The core transcriptional regulatory networks of chicken phGCs, chicken poGCs, mouse poGCs and human ovaries. Gene names of Hub-TFs (purple) are tagged.

To compare the core transcriptional regulators among species in GCs, we identified super-enhancers in chicken phGCs (pre-hierarchical granulosa cells), chicken poGCs (preovulatory granulosa cells), mouse poGCs (Dinh et al., 2019), and human ovaries (Consortium, 2012; Davis et al., 2018) based on H3K27ac histone modification data. A total of 476, 386, 423, and 322 super-enhancers were identified among chicken phGCs, chicken poGCs, mouse poGCs, and human ovaries, respectively (Supplementary Table S5). Enhancer or super-enhancer signals were detected near hormone receptor genes affecting follicle development and were highest near FSHR in chicken phGC, AR in chicken poGC, and ESR2 in mouse poGC compared with other hormone receptor genes (Figure 4A; Supplementary Figure S5A, no enhancer signals were detected near hormone receptors in the human ovary). The pathway enrichment analysis showed that super-enhancer associated genes (SEGs) were mainly enriched in follicle development-related pathways, such as the TGF beta signalling pathway, MAPK signalling pathway, and PI3K-Akt signalling pathway (Figure 4B; Supplementary Figure S6A), suggesting that these SEGs are associated with follicular development. To compare the core transcriptional regulators among species, the core transcriptional regulatory network of each species was identified based on super-enhancers (see Supplementary File S1 Methods for details). Closeness centrality (out) was used to assess the external regulatory capacity of transcription factors, whereas closeness centrality (in) was the opposite. Based on the closeness centrality (out) of each node in the network (Supplementary Figure S6B), a set of hub transcriptional regulatory factors (hub-TFs) (chicken phGCs 21, chicken poGCs 20, mouse poGCs 34, and human ovaries 34, details in Supplementary Table S5 with sheet name “hub-TFs Categories”) were identified (Figure 4C, purple point), of which AR with the highest closeness centrality (out) was a hub-TF only in chickens (Supplementary Table S5 with sheet name “ph SEGs closeness centrality” and “po SEGs closeness centrality”; Figure 4C, top of the graphic). Most hub TFs belonged to transcriptional regulatory circuits (chicken phGCs 100.00%, chicken poGCs 90.00%, mouse poGCs 73.53%, and human ovaries 61.76%) (words in blue in Supplementary Table S5 with sheet name “hub-TFs Categories”), suggesting that hub TFs maybe key transcriptional regulator factors for GCs (Hnisz et al., 2013; Whyte et al., 2013).

### Inhibition and Agitation of Androgen Receptors *In Vivo* Results in Abnormal Follicle Selection and Ovulation in Chickens

**FIGURE 5:**
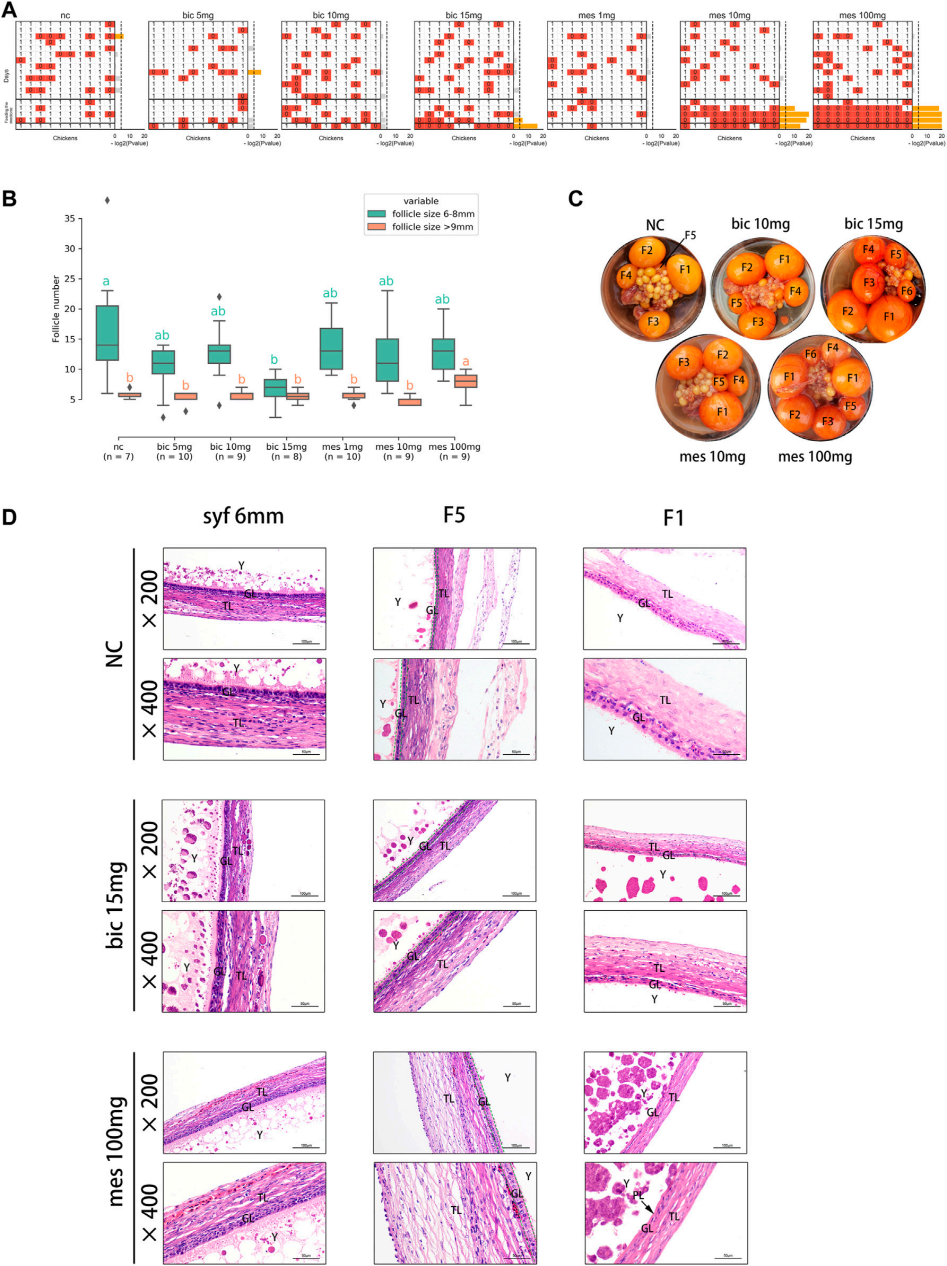
Inhibition and agitation of androgen receptors *in vivo* results in abnormal follicle selection and ovulation in chickens. **(A)** Effects of different doses of drugs on egg production. 0, no egg production; 1, egg production. *p*-values were calculated using the binomial test. *p* ≤ 0.05 is marked in yellow. **(B)** Effects of different doses of drugs on the number of pre-hierarchical and preovulatory follicles in chickens. Multiple comparisons were applied to each group in pre-hierarchical and preovulatory follicles. **(C)** Effect of different doses of the drug on follicle histology. The hierarchies of preovulatory follicles are labelled (see *Methods* for detail). **(D)** Effect of different doses of the drug on the histology of the chicken follicle wall. All tissue sections were stained with haematoxylin and eosin. TL, theca cell layer; GL, granulosa cell layer; Y, yolk; PL, perivitelline membrane.

To validate the effects of AR on follicle development in chickens, the AR antagonist bicalutamide (bic, inhibition of the transcriptional activity of AR) (Masiello et al., 2002) and the agonist mestanolone (mes, an anabolic–androgenic steroid) (Arnold et al., 1963; Schanzer, 1996) were utilized in in vivo experiments, and egg-laying, follicle number, and follicular wall histomorphology were examined. The results showed that egg production decreased significantly after feeding on 15 mg bic, 10 mg mes, and 100 mg mes (Figure 5A). Multiple F1 follicles were found in the 100 mg mes group, possibly a manifestation of polycystic follicle syndrome (Figure 5C; Supplementary Figure S7A). A comparison of the numbers of follicles in syf (6–8 mm) and po (>9 mm) in each treatment (Figure 5B) showed that, compared with the NC group, the number of syf was significantly decreased in the 15 mg bic group, and the number of po was significantly increased in the 100 mg mes group. In follicle wall histomorphology, the granulosa layer (GL) and theca layer (TL) of syf 6 mm follicles did not change significantly across all groups (Figure 5D; Supplementary Figure S7B); the GL of F5 follicles increased to two layers in the 15 mg bic group (Figure 5D, middle of the graphic, marked with a green dotted line); the perivitelline layer (PL) of F1 follicles was thickened in the 10 mg mes and 100 mg mes groups compared with the other groups (Figure 5D; Supplementary Figure S7B). These results showed that 15 mg bic inhibited follicle selection and ovulation, 10 mg mes inhibited ovulation and 100 mg mes inhibited ovulation and caused a phenomenon similar to polycystic follicle syndrome.

### Effects of Inhibition and Agitation of Androgen Receptors on Biological Processes Related to Follicular Development

**FIGURE 6:**
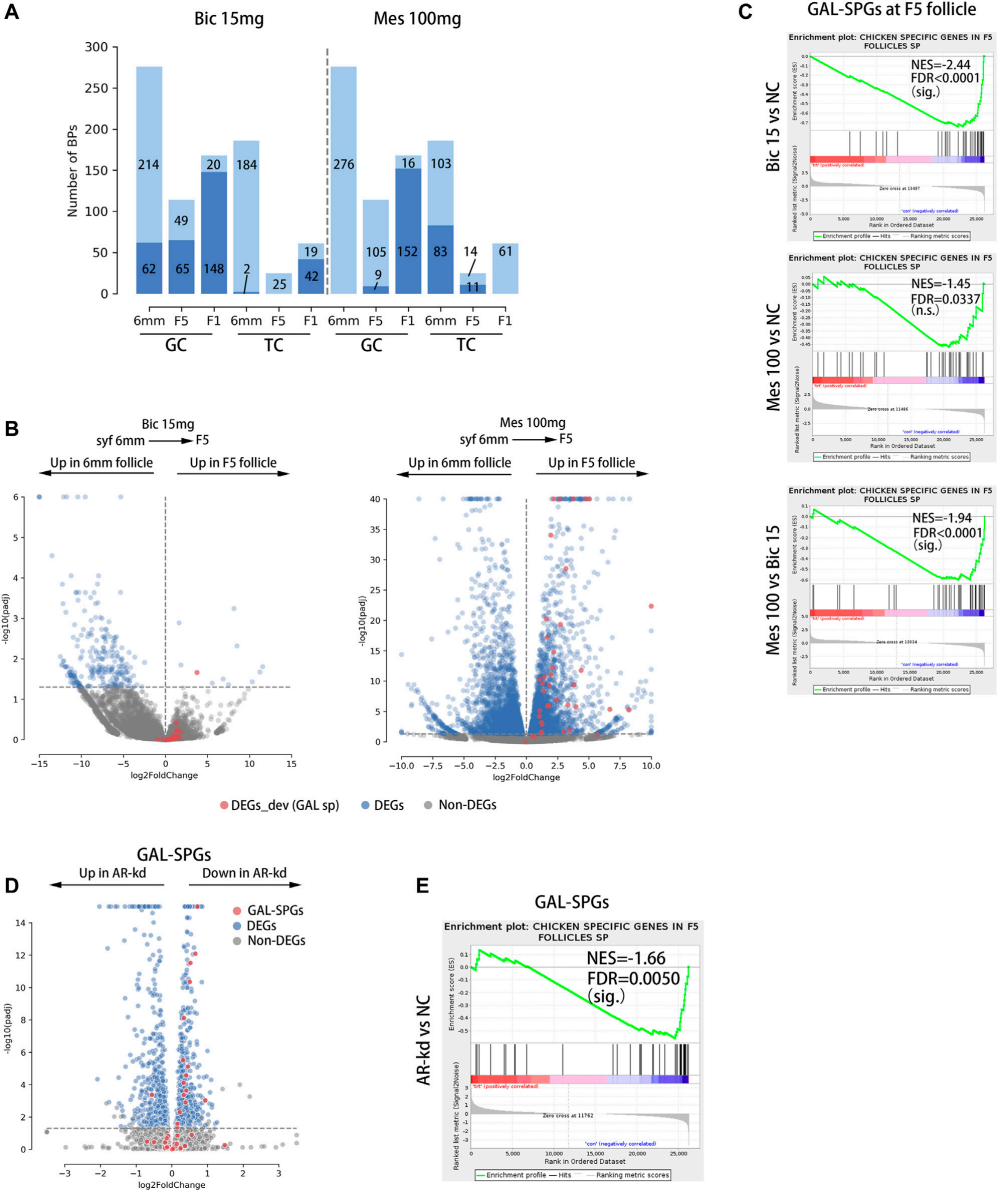
GAL-SPGs are essential for follicle selection in chickens. **(A)** Stacked bar plot showing the distribution of follicle development-related BPs that are significantly altered by inhibiting or enhancing the function of androgen receptors, which are in deep blue. The follicle development-related BPs that are not significantly altered by inhibiting or enhancing the function of androgen receptors are in light blue. The number of BPs in each group is labelled. **(B)** Volcano plot showing the differential expression of GAL-SPGs (red) before and after follicle selection (syf 6 mm vs F5) when AR function is inhibited (bic 15 mg, left of the graph) or enhanced (mes 100 mg, right of the graph). Differentially expressed genes (FPKM ≥ 1 and Padj ≤ 0.05) are marked in blue. **(C)** The GSEA results showing the consistent differences in GAL-SPGs at the F5 stage when AR function is inhibited (bic 15 mg, top of the graph) or enhanced (mes 100 mg, middle of the graph) as well as the consistent differences in GAL-SPGs at the F5 stage between mes 100 mg and bic 15 mg sig. *p* ≤ 0.01, n.s. *p* > 0.01. **(D)** Volcano plot showing the differential expression of GAL-SPGs (red) when AR was knocked down in poGCs. Differentially expressed genes (FPKM ≥ 1 and Padj ≤ 0.05) are marked in blue. **(E)** The GSEA results showing the consistent differences in GAL-SPGs when AR was knocked down in poGCs. sig. *p* ≤ 0.01, n.s. *p*＞0.01.

To investigate the effects of inhibition and agitation of ARs on biological processes related to follicular development, RNA-Seq was performed on GCs and TCs at three stages of follicular development (syf 6 mm, F5, F1) in the 15 mg bic and 100 mg mes groups (Supplementary Table S1), in which the phenotypes were highly variable (Supplementary Table S8). A gene set enrichment analysis (GSEA) was utilized to find significantly altered follicle development-related biological processes in GCs and TCs when AR was inhibited or agonized. As a result, 15 mg bic mainly down-regulated the biological processes related to follicular development in GCs at the syf 6 mm stage (22.46%), F5 stage (57.02%) and F1 stage (88.10%) and in TCs at the F1 stage (68.85%) (Figure 6A, left side of the dotted line, see Supplementary Figure S8 for detail BPs); 100 mg mes mainly down-regulated the biological processes related to follicular development in GCs at the F1 stage (90.48%) and in TC at the syf 6 mm stage (44.62%) and F5 stage (44.00%) (Figure 6A, right side of the dotted line, see Supplementary Figure S8 for detail BPs). These results show that 15 mg bic mainly affects biological processes related to follicular development in GCs, whereas 100 mg mes mainly affects biological processes related to follicular development in TCs.

### GAL-SPGs Are Essential for Follicle Selection in Chickens

To determine whether GAL-SPGs affect the follicle selection process, we compared the expression of GAL-SPGs in the 15 mg bic groups (follicle selection was inhibited) and 100 mg mes groups (follicle selection was normal) before and after follicle selection (syf 6 mm and F5 stages). As a result, the expression of GAL-SPGs in the 15 mg bic group was not significantly different (*p*adj ≤ 0.05) before and after follicle selection (Figure 6B, left of the graphic), whereas the expression of GAL-SPGs in the 100 mg mes group was significantly highly expressed (*p*adj ≤ 0.05) after follicle selection (Figure 6B, right of the graphic), which was consistent with the expression profile of GAL-SPGs in the normal group (Supplementary Figure S4A), indicating that GAL-SPGs are required for follicle selection in chickens. To determine the effect of ARs on SPGs, we compared the expression of GAL-SPGs in GCs at the F5 stage in the normal group, the 15 mg bic group and the 100 mg mes group, as both the phenotypic changes of abnormal follicle selection (Figure 5D, middle of the graphic, marked with a green dotted line) and GAL-SPGs (Figure 3A, upper middle of the graphic) were found in GCs at the F5 stage. The results showed that, compared with the normal group, inhibition of ARs (15 mg bic group) significantly down-regulated the expression of GAL-SPGs (Figure 6C, top of the graphic), agitation of ARs (100 mg mes group) had no significant effect on the expression of the GAL-SPGs (Figure 6C, middle of the graphic), and the GAL-SPGs were significantly down-regulated in the 15 mg bic group compared with the 100 mg mes group (Figure 6C, bottom of the graphic), suggesting that inhibition of ARs may down-regulate the expression of GAL-SPGs. To verify this hypothesis, AR was knocked down in the chicken poGCs cultured *in vitro*, and the expression of GAL-SPGs was examined (Supplementary Tables S1, S9). The results showed that knockdown of AR (Supplementary Figure S9A) significantly promoted the proliferation of GCs (Supplementary Figure S9B), which was consistent with the inhibition of AR inducing an increase in the number of GLs at the F5 stage in the *in vivo* experiment (Figure 5D, middle of the graphic, marked with green dotted lines). Compared with the normal group, 43.59% (Bahr et al., 1983) of GAL-SPGs were significantly down-regulated in AR-kd poGCs, whereas only one GAL-SPG was significantly up-regulated in AR-kd poGCs (Figure 6D). The GSEA results showed that GAL-SPGs were significantly down-regulated in AR-kd poGCs compared with the normal group (Figure 6E). These results indicate that knockdown of ARs significantly down-regulates the expression of GAL-SPGs. Thus, inhibition of ARs significantly reduces the expression of GAL-SPGs, which in turn prevents follicular selection.

### The AR Super-Enhancer Genomic Region in Chicken poGCs Is Specific to Birds

**FIGURE 7:**
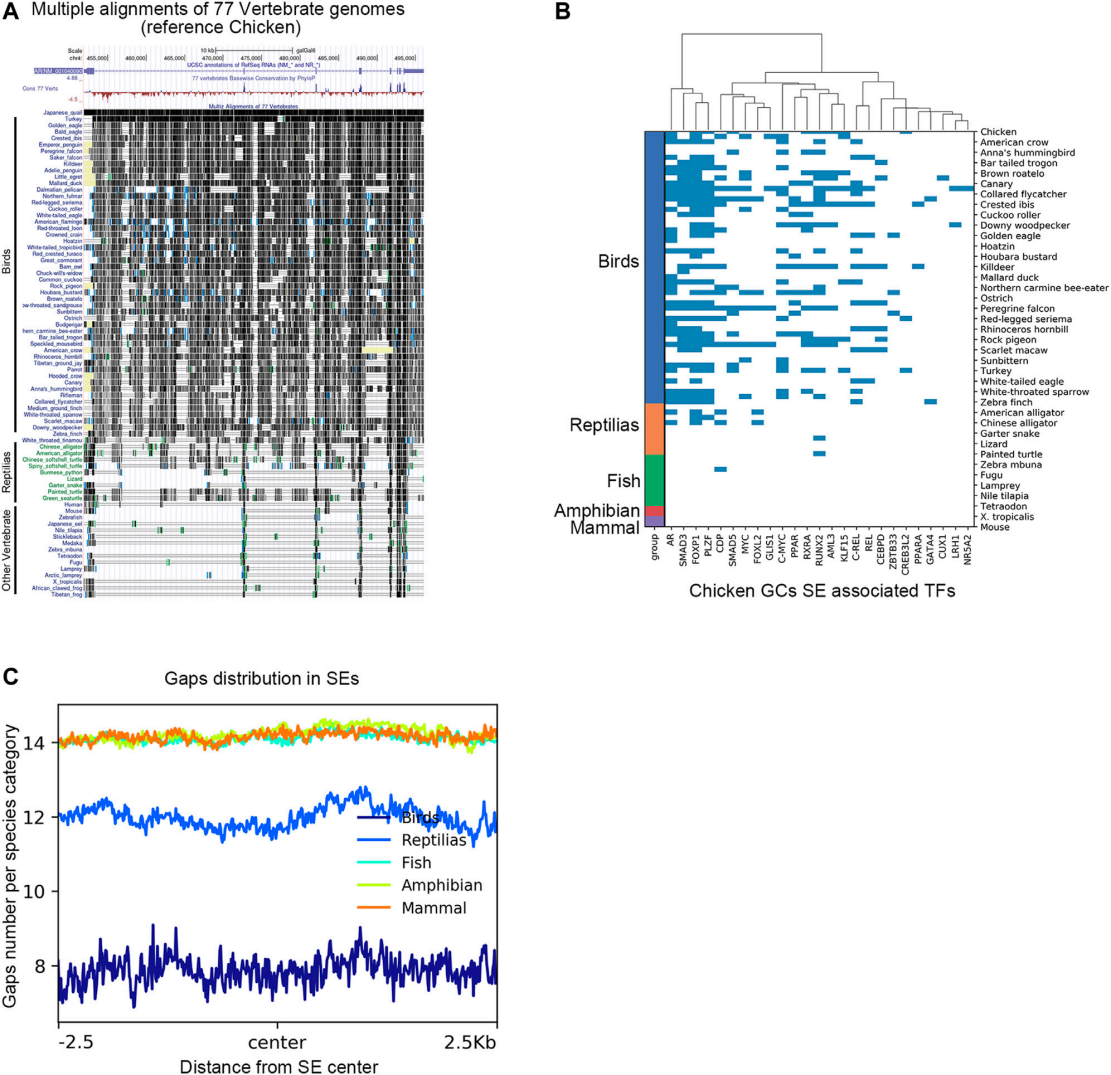
The AR super-enhancer genomic region in chicken poGCs is specific to birds. **(A)** Multiple alignments of the 77 vertebrate genomes in the UCSC Genome Browser at the AR locus using chickens as a reference. **(B)** Binding profile of candidate TFs screened by chicken poGC super-enhancers in genomic regions homologous to the chicken AR super-enhancer in 77 species. **(C)** Gaps distribution in genomic regions homologous to chicken poGC super-enhancers across 77 species.

To characterize the genomic region of AR super-enhancers in chicken poGCs across species, we compared genomic regions homologous to chicken AR super-enhancers across species based on multiple sequence alignment results from the UCSC Genome Browser. In the multiple sequence alignment results of 77 vertebrate genomes (with the chicken genome as referenced), the AR super-enhancer region-specific to chicken poGCs is remarkably conserved in the bird genome, and the corresponding homologous fragments of other species contain a large number of deletions (Figure 7A). Genomic sequence divergence may alter transcription factor binding sites in super-enhancer regions; therefore, candidate TFs screened by chicken poGC super-enhancers were used to explore their binding profile in genomic regions homologous to the chicken AR super-enhancer in 77 species (Figure 7B). These TFs were heavily bound in the AR super-enhancer homologous region of birds, partially bound to the AR super-enhancer homologous region of reptiles, and barely bound to the AR super-enhancer homologous region of other species. Moreover, we explored the gap distribution in genomic regions homologous to chicken poGC super-enhancers across 77 species and found that the homologous regions of all species except birds and reptiles (mammals, amphibians, and fish) contained many gaps (Figure 7C), implying that the whole super-enhancer regions in chicken GCs are generally bird-specific.

## Discussion

In this study, we matched the correspondence of follicle development stages among chickens, humans, and cows and compared the similarities and differences in follicular development-related genes and related biological processes among species (Table 1), and on this basis identified chicken-specific follicle development-related genes. The comparison of core transcriptional regulators in GCs among species revealed that AR was a core transcriptional regulator specific to chicken GCs. Inhibition of AR in both *in vivo* and *in vitro* experiments significantly down-regulated GAL-SPGs and results in abnormal follicle selection. Therefore, AR can regulate follicle selection through GAL-SPGs.

**TABLE 1:** Similarities and differences of GC and TC among species.

Item	Similarities	Differences
Sex hormone genes	The expression trends of androgen receptors in both GCs and TCs of chicken follicles were opposite to those in human and bovine	
Genes related to follicle development	Follicle development-related genes in both chicken follicle GCs and TCs are specific among species	
Distribution of genes related to follicle development		Follicle development-related genes were mainly expressed in GCs compared to TCs
Biological processes related to follicular development	1. Biological processes related to cell proliferation and differentiation predominate in both GCs and TCs before follicle selection	1. In TCs before follicle selection, the chicken-specific biological processes associated with follicle development are mainly associated with mitosis
	2. Biological processes related to signal transduction, substance transport and tissue development predominate in both GCs and TCs after follicle selection	2. In GCs after follicle selection, the chicken-specific biological processes associated with follicle development mainly include substance transport and hormone signaling
		3. In TCs after follicle selection, the chicken-specific biological processes associated with follicle development are mainly signal transduction, which includes BMP and SMAD signalling pathways important for follicle development

### Biological Processes Associated With Follicle Development Specific to Chickens

Our results showed that the genes and biological processes associated with follicle development at each stage are more species-specific (Figures 2A, 3A), where chicken-specific biological processes are distinct before and after follicle selection (selected and differentiated stages) (Supplementary Table S4 with sheet name “BPs specific to chickens”). The biological processes specific to chickens in TCs at the selected stage are mainly associated with mitosis (Supplementary Table S4 with sheet name “BPs specific to chickens”), suggesting that, unlike mammals, TCs may also proliferate rapidly in chickens during follicle development, allowing the follicle to protrude outside the ovarian stroma, resulting in the unique mulberry-like structure of the chicken ovary. The biological processes specific to chickens in GCs at the differentiated stage are mainly associated with substance transport and hormone-related signalling pathways (Supplementary Table S4 with sheet name “BPs specific to chickens”), suggesting that after follicle selection, chicken GCs maybe influenced by hormones activating more substance transport processes to accumulate more yolk material. The biological processes specific to chickens in TCs at the differentiated stage are mainly associated with signal transduction (Supplementary Table S4 with sheet name “BPs specific to chickens”), which contain BMP- and SMAD-associated signalling pathways important for follicle development (Sánchez and Smitz, 2012), indicating that some biological processes important for follicle development in mammalian GCs and oocytes occur in chicken TCs. The genes CYP19A1 and ESR2, which are important for follicle development, are also expressed in chicken TCs (Wang and Gong, 2017), suggesting the important role of chicken TCs in follicle development.

### The Potential Role of GAL-SPGs in Chicken Follicle Development

We identified chicken-specific follicle development-related candidate genes (GAL-candSPGs) in GCs at the differentiation stage (diffGCs) and in TCs at the differentiation stage (diffTCs) based on genes in galBPs-diffGCs and galBPs-diffTCs (Figure 3B; Supplementary Figure S3A bottom of the graphic). As only the GAL-candSPGs and their associated biological processes in diffGCs were specific to chickens, the GAL-candSPGs in diffGCs were identified as GAL-SPGs.

GAL-SPGs were mainly enriched in transport-related biological functions, which contain a large number of transmembrane transport proteins, such as the solute carrier (SLC) family and potassium channel (KCN) family (Supplementary Figure S4D). A previous study in mice showed that oocytes promoted the uptake of l-alanine and l-histidine by CGCs by elevating the expression level of Slc38a3 in CGCs, which in turn promoted oocyte maturation, suggesting that the transport process of substances involved in transmembrane proteins maybe important for oocyte maturation (Eppig et al., 2005). During follicle development, the size of preovulatory follicles in chickens vary dramatically, growing from 9 to 30 mm in 7 days (Lovell et al., 2003), whereas human follicles take 10 days to grow from 10 to 20 mm (Gougeon, 1986), suggesting that GAL-SPGs maybe responsible for the rapid accumulation of yolk material in chicken follicles after selection. We therefore hypothesized that the expression of GAL-SPGs increases dramatically in selected follicles during chicken follicle development (Supplementary Figures S4A–C) and is maintained in preovulatory follicles, thereby promoting oocyte maturation and yolk material accumulation, allowing unselected follicles to enter the preovulatory stage and gradually mature.

### The Role of AR in Chicken Follicle Development

A study in mice has shown that knocking out AR results in reduced numbers of healthy follicles and reduced pup production in mice with high fecundity (24–32 weeks) (Shiina et al., 2006). Low doses of androgens (DHT, dihydrotestosterone) can enhance ovulation in mice, whereas high doses of androgens cause polycystic follicle syndrome (Sen et al., 2014; Caldwell et al., 2017). In our study, inhibition of AR significantly reduced the number of selected follicles (6–8 mm) in chickens and terminated ovulation, whereas agitation of AR caused symptoms similar to polycystic follicle syndrome (Figure 5), suggesting that AR is important for follicle development in both oviparous and viviparous animals. A comparison of core transcriptional regulators of GCs among species showed that AR is a core transcriptional regulator specific to chicken GCs (Figure 4) and that androgen and its receptor content tended to increase during follicular development in chickens (Supplementary Figure S1), whereas it tended to decrease in humans (Kristensen et al., 2017), suggesting that the role of AR in follicular development in chickens may differ from that in mammals. The results of AR inhibition in both *in vivo* and *in vitro* experiments indicated that inhibition of androgen receptors significantly down-regulates GAL-SPGs and inhibits follicle selection processes (Figure 6), suggesting that AR may affect follicle selection by regulating these follicle development-related genes specific to chickens. Thus, AR may promote the expression of chicken-specific transmembrane proteins by elevating GAL-SPGs, which in turn promote the accumulation of yolk material and oocyte maturation, facilitating follicle selection. Research in polycystic follicular syndrome has found that neuroendocrine androgen action is a key extra ovarian mediator in the development of polycystic ovary syndrome (Caldwell et al., 2017), and the role of extra ovarian AR in follicle development cannot be ignored.

### The Role of AR in Follicle Selection Maybe Widespread in Birds

A previous study showed that different phenotypes between species are associated with genomic divergence in species-specific noncoding regulatory elements (Han et al., 2018). The chicken AR super-enhancer was established in avian-specific genomic regions and the binding pattern of the chicken AR super-enhancer to core transcription factors was also bird-specific, suggesting that the specific role of AR in follicle selection maybe widespread in birds. In evolution, the transition from oviparity to viviparity was accompanied by an increase in ESR2 expression (Gao et al., 2019). Our study suggests that the role of AR in the transition from oviparity to viviparity should be considered.

## Conclusion

In conclusion, inhibition of chicken AR down-regulates the expression of GAL-SPGs, which in turn prevents follicle selection. Thus, AR regulates follicle selection through a set of chicken-specific follicle development-related genes, in which changes in the expression of GAL-SPGs may affect the expression levels of transmembrane transporter proteins that are responsible for the accumulation of yolk material during chicken follicle selection. Our findings suggest that AR is important for follicle development in chickens and can explain the phenomenon of differential hormone content between chickens and mammals during follicle development, providing a new perspective to understand the process of follicle development in chickens. Our research suggests that androgens can be used in practice to control the egg-laying process, e.g. by adding androgens in small amounts to stabilise high egg production rates in chickens. Based on our study, researchers can further explore a range of issues such as the loci in the chicken-specific AR super-enhancer associated with chicken egg-laying traits, the transcriptional regulatory complexes bound at the chicken-specific AR super-enhancer, and the specific functions of the chicken-specific follicle development-related genes.

## Materials and Methods

### Animals and Treatments

The experiments performed in this research were approved by the Ethics Committee of Huazhong Agricultural University. All experimental hens (35–40 weeks of age) were obtained from Hubei *Xintianmu* Agricultural Science and Technology Development Co. Ltd. (*Hubei*, China). For the androgen receptor-related drug experiment, chickens were randomly divided into seven groups (10 birds/group). The androgen receptor antagonist-treated groups (three groups) were fed 5, 10, and 15 mg of bicalutamide (AbMole, M1960); the androgen agonist-treated groups (three groups) were fed 1, 10, and 100 mg of mestanolone (AbMole, M3748); and the blank group (one group) was fed water as a control. All drugs were fed for 4 days, and follicles were collected after 1 day of drug withdrawal. All hens were freely fed and watered.

The binom_test function of Scipy (v1.3.1) was used to perform a binomial test to determine whether there was a significant difference in the number of eggs laid in each group before and after drug feeding. The parameter p of binom_test is the mean egg-laying rate for each group before feeding (14 days), alternative = “less”. *p* ≤ 0.05 was considered significant.

### Labelling of Preovulatory Follicles

The hierarchy of preovulatory follicles was labelled according to a previously described method (Johnson, 2015). Follicles larger than 9 mm with visible vascular structures are defined as preovulatory follicles. The average number of preovulatory follicles in chickens is 5 to 6; therefore, they are generally labelled F5/F6-F1 in descending order of follicle size. In this study, the number of follicles before ovulation in chickens was more than 6 due to the influence of AR drugs; therefore, after labelling follicles as described above, all unlabelled follicles were labelled F1.

### Hormone Radioimmunoassay

The testosterone content of the chicken follicle wall and follicular fluid was determined according to a previously described experimental method (Li et al., 2019).

### Isolation of Chicken Follicular Granulosa Cells and Theca Cells

Granulosa and theca cells were isolated from fresh follicular tissue of hens according to a previously described experimental method (Gilbert et al., 1977; Jing et al., 2018).

### Follicular Granulosa Cell Culture and Treatment

Establishment of a granulosa cell culture and siRNA-mediated knockdown of the androgen receptor in granulosa cells were performed according to previously described methods (Luo et al., 2020). The target sequence of AR-siRNA is GGT​TGG​AGA​TCT​TTC​ACT​A.

### Chromatin Immunoprecipitation (ChIP) of Chicken Follicle Granulosa Cells and Library Preparation

According to a previous description (Wang et al., 2019), ChIP experiments with histone-modified H3K27ac (Abcam, ab4729) were performed in pre-hierarchical and preovulatory granulosa cells were cultured for 8 h, and ChIP DNA libraries were constructed. The ChIP DNA library was sent to Novogene Co., Ltd. (Beijing, China) for paired-end 150 bp sequencing on the Illumina Nova platform.

### RNA Library Construction and Sequencing of Chicken Follicle Granulosa and Theca Cells

Tissues from follicular granulosa and theca cell layers from hens (three biological replicates) of blank (NC), 15 mg bicalutamide (bic 15 mg), and 100 mg mestanolone (mes 100 mg) were sent to Novogene Co., Ltd. (Beijing, China) for RNA extraction, library construction, and sequencing. All samples underwent paired-end 150 bp sequencing on the Illumina Nova platform.

### Follicular Histology Assessment

Follicular tissues were fixed with 4% paraformaldehyde and embedded in paraffin, and 5-μm sections were cut out for haematoxylin and eosin staining.

### Data Processing

See Supplementary File S1.

### General Statistics and Plots

Unless otherwise stated, all multiple comparisons were first performed with one-way ANOVA by using the aov function in R software. Significantly different data were then subjected to multiple comparisons using the LSD.test function of agricolae (v1.3.3) and marked with an alphabetical symbol for significant differences, with *p* ≤ 0.05 considered significant. All logarithmic scaling of data was performed using the log2 function of Numpy (v1.18.1) in Python (v3.7). All data normalization (Z-normalization, polar normalization) was performed using StandardScaler and MinMaxScaler from scikit-learn (v0.20.3). All mapping was performed using the Matplotlib (v3.1.1) and Seaborn (v0.9.0) libraries in Python (v3.7). Transcriptional regulatory network diagrams were drawn using bundle_graph from HoloViews (v1.12.7).

## Data Availability

ChIP-seq and RNA-Seq data produced in this study have been submitted to the NCBI Gene Expression Omnibus (GEO; https://www.ncbi.nlm.nih.gov/geo/) under accession number GSE163891. The in-house scripts used in this research are freely available for download from GitHub (https://github.com/yingHH/in-house_scripts_for_paper-20201228) or Gitee (https://gitee.com/github-HY/in-house_scripts_for_paper-20201228).

## Supplementary Material

Supplementary Figure S1 Expression of hormones and hormone receptors during follicular development among species. **(A)** Hormone levels in the follicle wall (top) and follicular fluid (bottom) during preovulatory follicular development in chickens. **(B)** Expression of hormone receptors during follicular development among species. Significance of differentially expressed genes calculated using DESeq2 (chicken and cow) and Seurat (human). Slct, selected stage; Diff, differential stage; Preo, preovulatory stage. *n* = 3, * *p* ≤ 0.05, n.s. *p* > 0.05. LogFC < 0 indicates that the gene is highly expressed at the po stage, and LogFC > 0 indicates that the gene is highly expressed at the slct stage.

Supplementary Figure S2 Distribution of gene expression of GCs and TCs in each follicle sample before and after removing the batch effect.

Supplementary Figure S3 Features of candidate chicken-specific BPs in galBPs-slctTCs and galBPs-diffTCs. **(A)** Candidate chicken-specific BPs in TCs at selected and differentiated stages. On the left of the graph are the top ten BPs specific to chickens in TCs at the selected or differentiated stage. On the right of the graph is a Venn diagram showing the overlap between galBPs-slctTCs (red) and all BPs in GCs (green) or between galBPs-diffTCs (red) and all BPs in GCs (green). The candidate chicken-specific BPs are marked in red. *p* was calculated using hypergeometric test, *p* ≤ 0.0001. Candidate chicken-specific BPs were only in galBPs-diffTCs, marked with red arrows, and these BPs were used to identify candidate chicken-specific follicle development-related genes. Previously reported follicle development-related genes are marked with red arrows. **(B)** Pie chart showing the percentage and corresponding gene names of candidate chicken-specific follicle development-related genes (red) identified from candidate chicken-specific BPs in galBPs-diffTCs.

Supplementary Figure S4 Features of GAL-SPGs. **(A)** Volcano plot showing the differential expression of GAL-SPGs (red) before and after follicle selection (syf 6 mm vs F5). Differentially expressed genes (FPKM ≥ 1 and *p*adj ≤ 0.05) are marked in blue. **(B)** The GSEA results showing the consistent differences in GAL-SPGs between syf with high and low FSHR expression. sig. *p* ≤ 0.01, n.s. *p* > 0.01. **(C)** Volcano plot showing the differential expression of GAL-SPGs (red) between syf with high and low FSHR expression. Differentially expressed genes (FPKM ≥ 1 and *p*adj ≤ 0.05) are marked in blue. **(D)** GO annotations for GAL-SPGs.

Supplementary Figure S5 IGV screenshots showing the H3K27ac ChIP-Seq binding profiles around hormone receptor genes. Super-enhancer regions are marked with red boxes, and enhancer regions identified by MACS2 are marked with orange boxes.

Supplementary Figure S6 Features of super-enhancers in chicken phGCs, chicken poGCs, mouse poGCs and human ovaries. **(A)** Top ten KEGG pathways of super-enhancer associated genes in chicken phGCs and chicken poGCs. **(B)** Distribution of closeness centrality (in) and closeness centrality (out) for each network node. Hub-TFs are in orange, and non-hub TFs are in blue.

Supplementary Figure S7 Effects of different doses of drugs on the histology of chicken follicles. **(A)** Effect of 100 mg mes on the histology of chicken follicles. The hierarchies of preovulatory follicles are labelled (see Methods for detail). **(B)** Effects of different doses of drugs on the histology of the chicken follicle wall. All tissue sections were stained with haematoxylin and eosin.

Supplementary Figure S8 Top ten follicle development-related BPs in each stage that are significantly altered by inhibiting or enhancing the function of androgen receptors.

Supplementary Figure S9 In vitro experiments to knock down AR in poGCs. **(A)** The qPCR results show that siRNA significantly reduced AR gene expression in GCs cultured in vitro at the preovulatory stage. *n* = 4, *** *p* ≤ 0.05. **(B)** In vitro proliferation experiments showed that knockdown of AR significantly promoted the proliferation of preovulatory follicular granulosa cells. *n* = 4, * *p* ≤ 0.05.

Supplementary Table S1 Summary of rawdata.xlsx.

Supplementary Table S2 Expression matrix of homologous genes of GCs and TCs.xlsx.

Supplementary Table S3 DEGs of GCs and TCs across stages among species.xlsx.

Supplementary Table S4 Follicle development related BPs among species.xlsx.

Supplementary Table S5 SEGs of GCs or ovary among species.xlsx.

Supplementary Table S6 Egg production records.xlsx.

Supplementary Table S7 Follicle Count Record.xlsx.

Supplementary Table S8 Gene expression matrix and DEGs of follicular GCs and TCs after AR drug stimulation.xlsx.

Supplementary File S1 Methods for data processing.docx.
