# Supplementary figures and images for: Comparative Analysis Among Different Species Reveals That the Androgen Receptor Regulates Chicken Follicle Selection Through Species-Specific Genes Related to Follicle Development
Source: Front Genet. 2022 Jan 3;12:752976. doi: 10.3389/fgene.2021.752976 (PMC8762282; doi:10.3389/fgene.2021.752976)

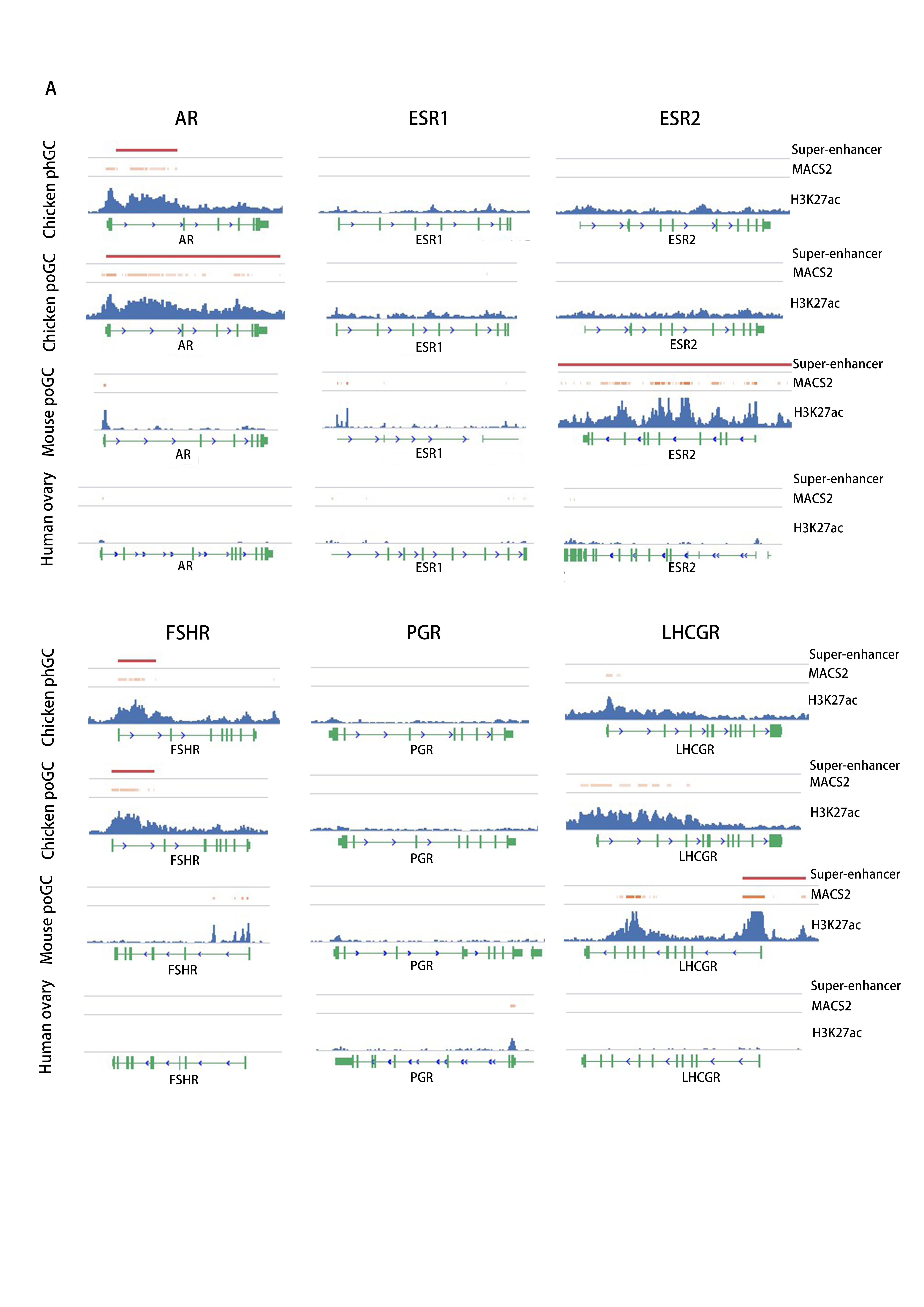

Supplement: Supplementary file 3 [file Image5.PNG]

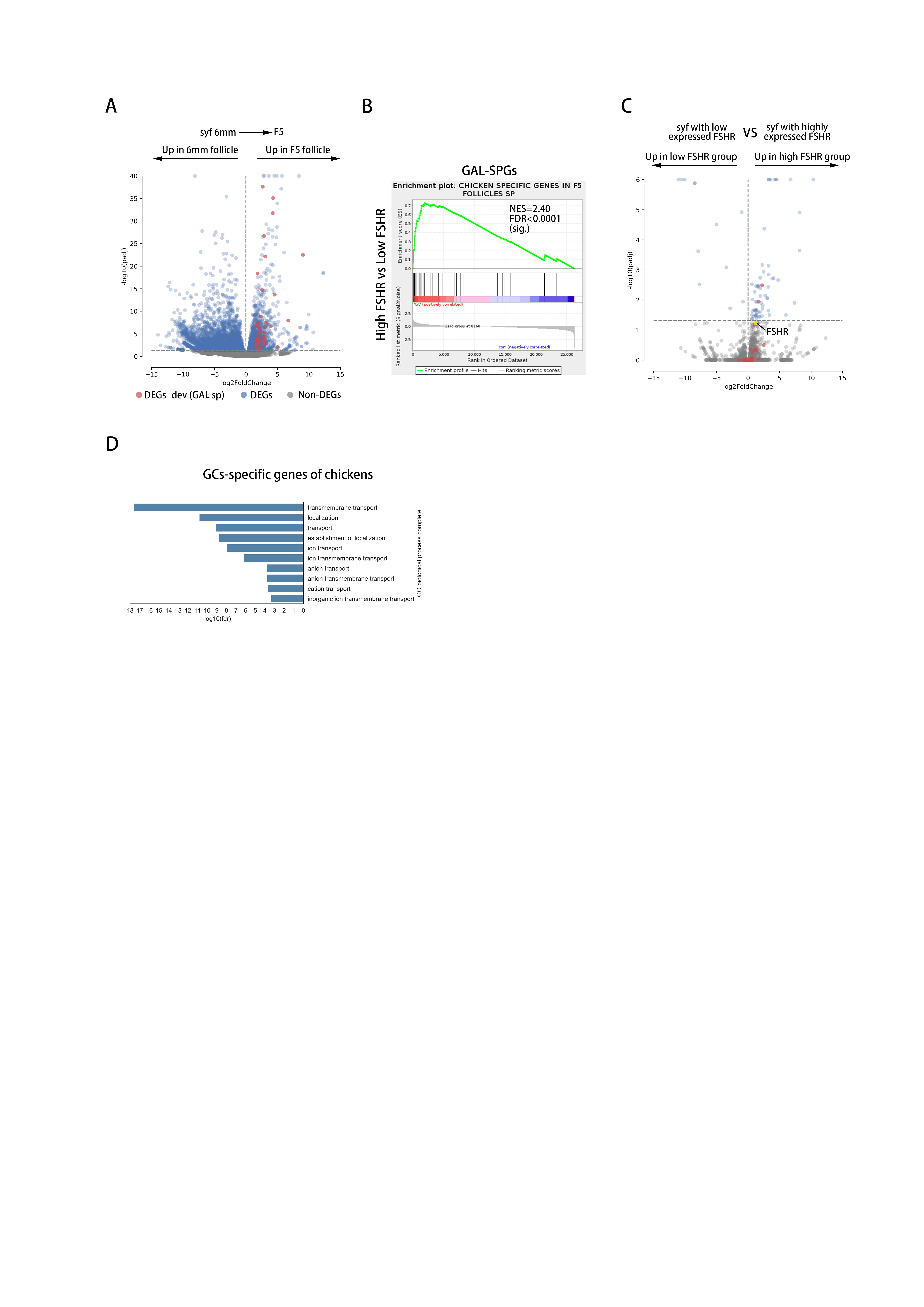

Supplement: Supplementary file 4 [file Image4.PNG]

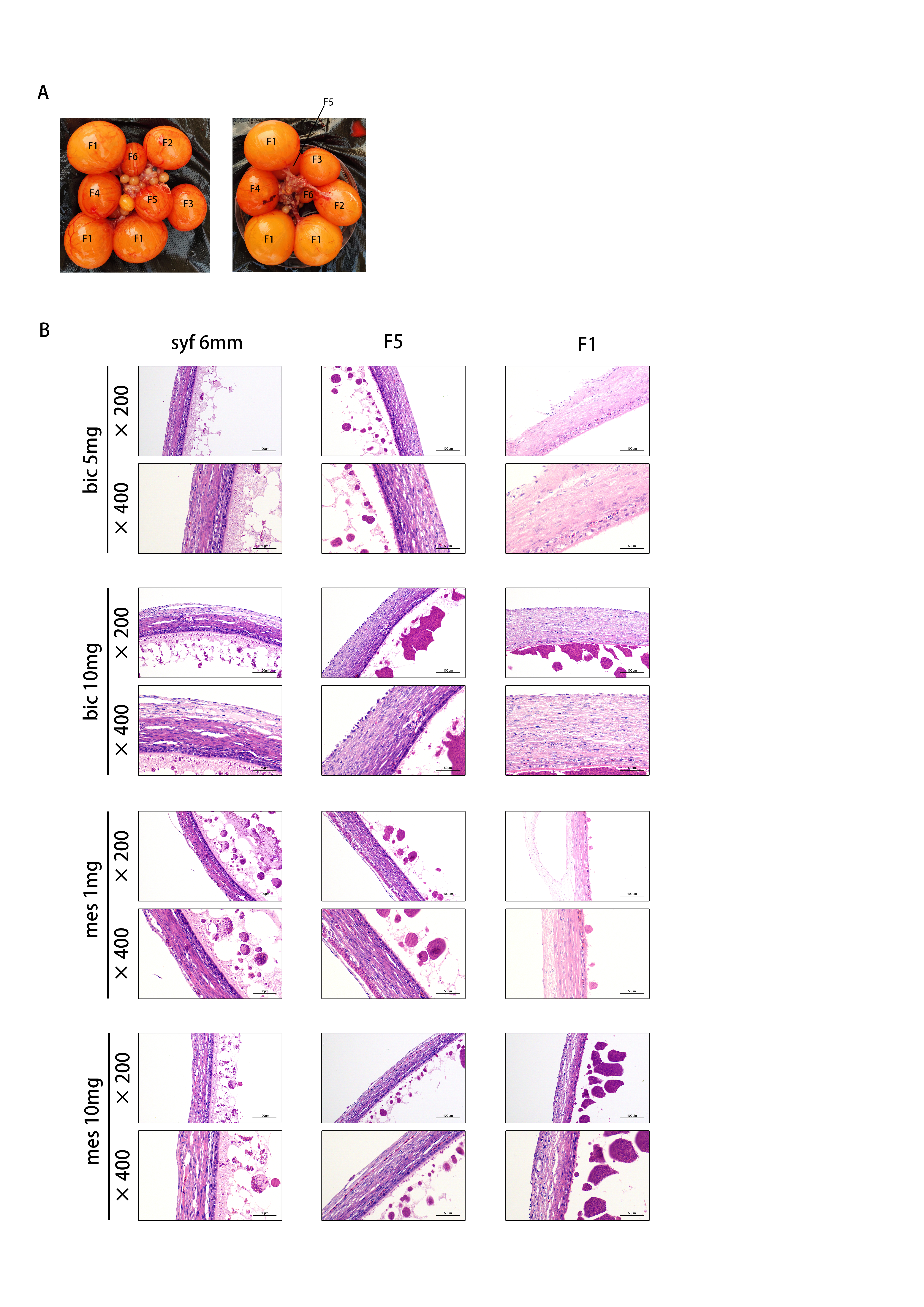

Supplement: Supplementary file 7 [file Image7.PNG]

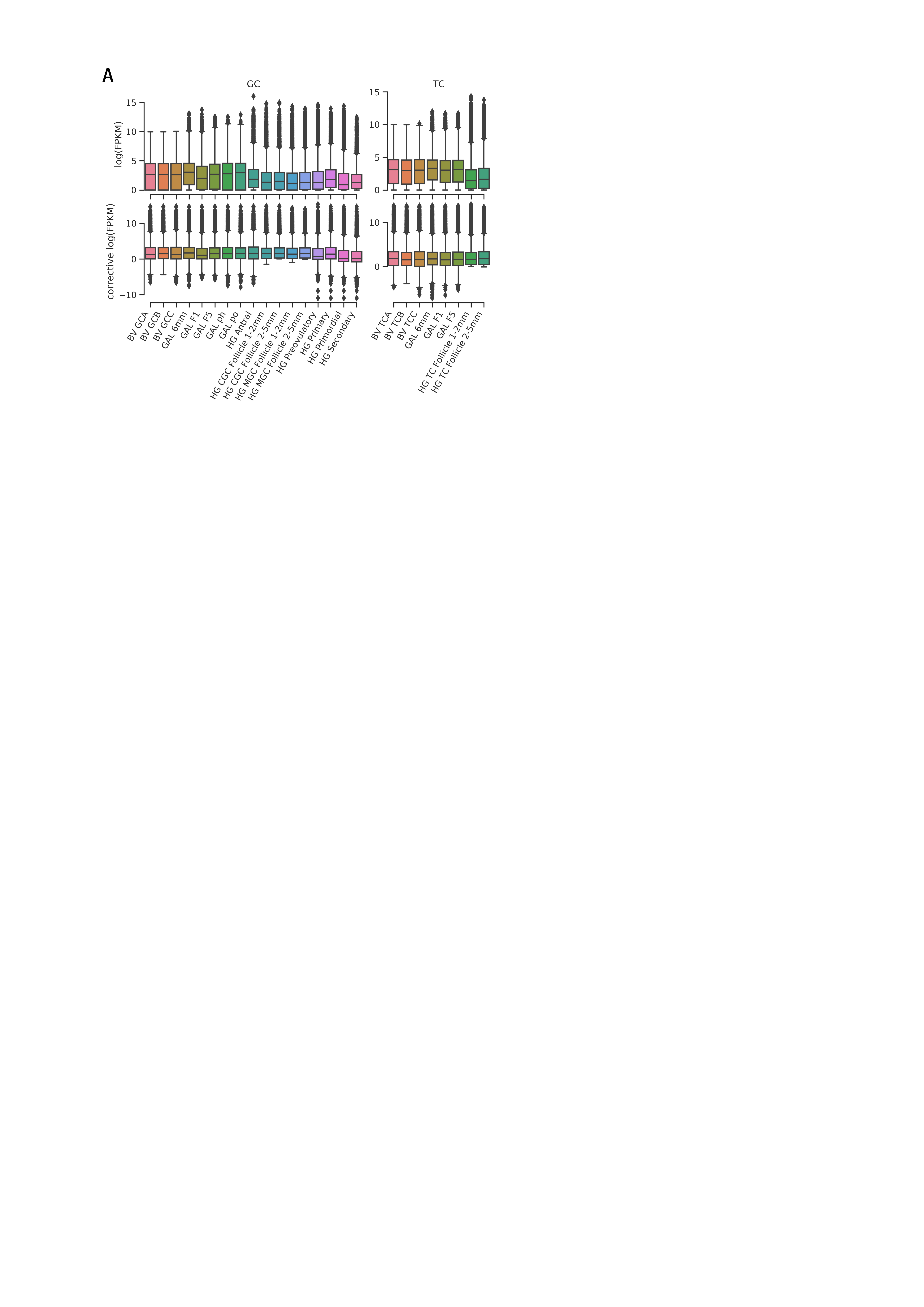

Supplement: Supplementary file 8 [file Image2.PNG]

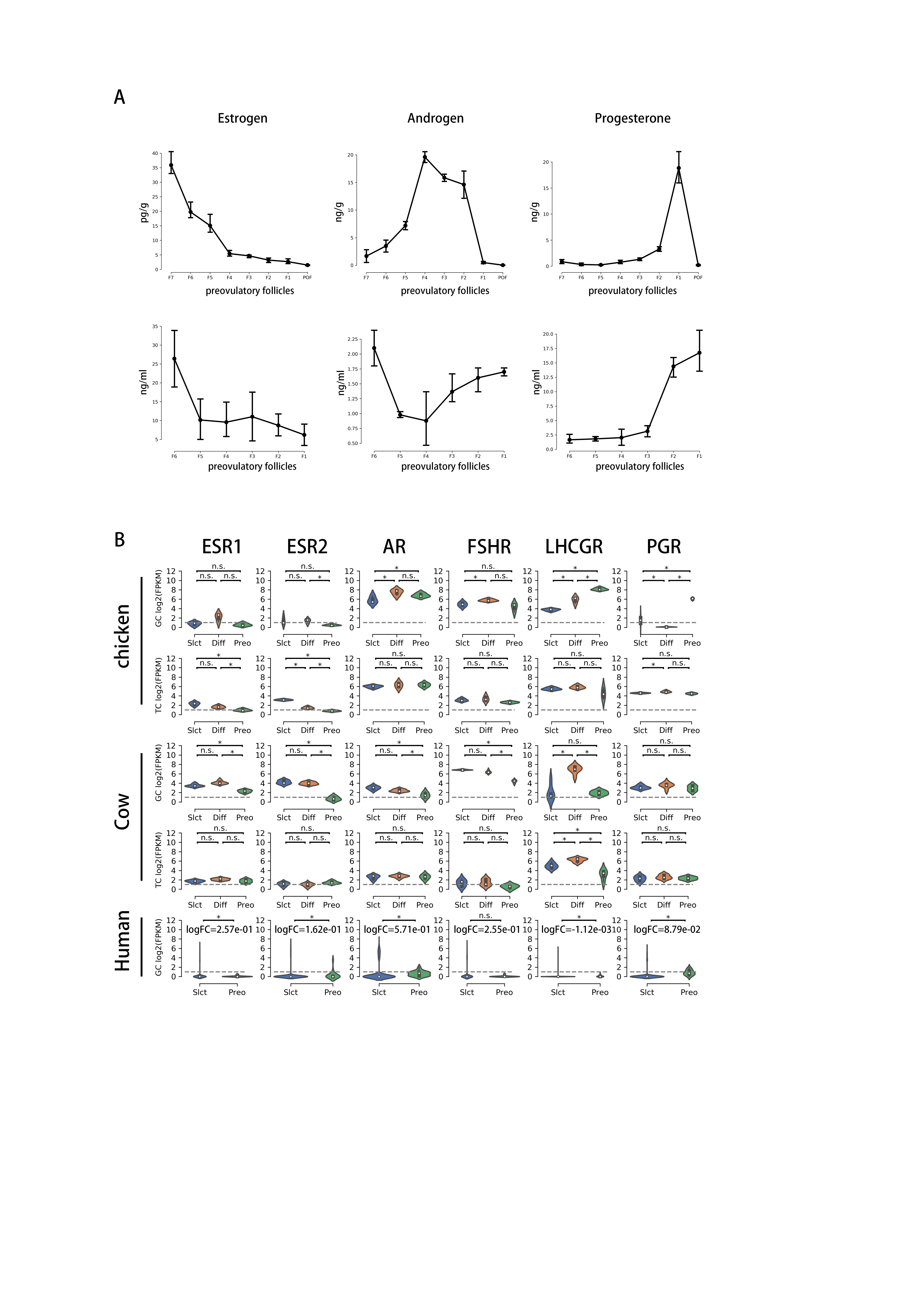

Supplement: Supplementary file 10 [file Image1.PNG]

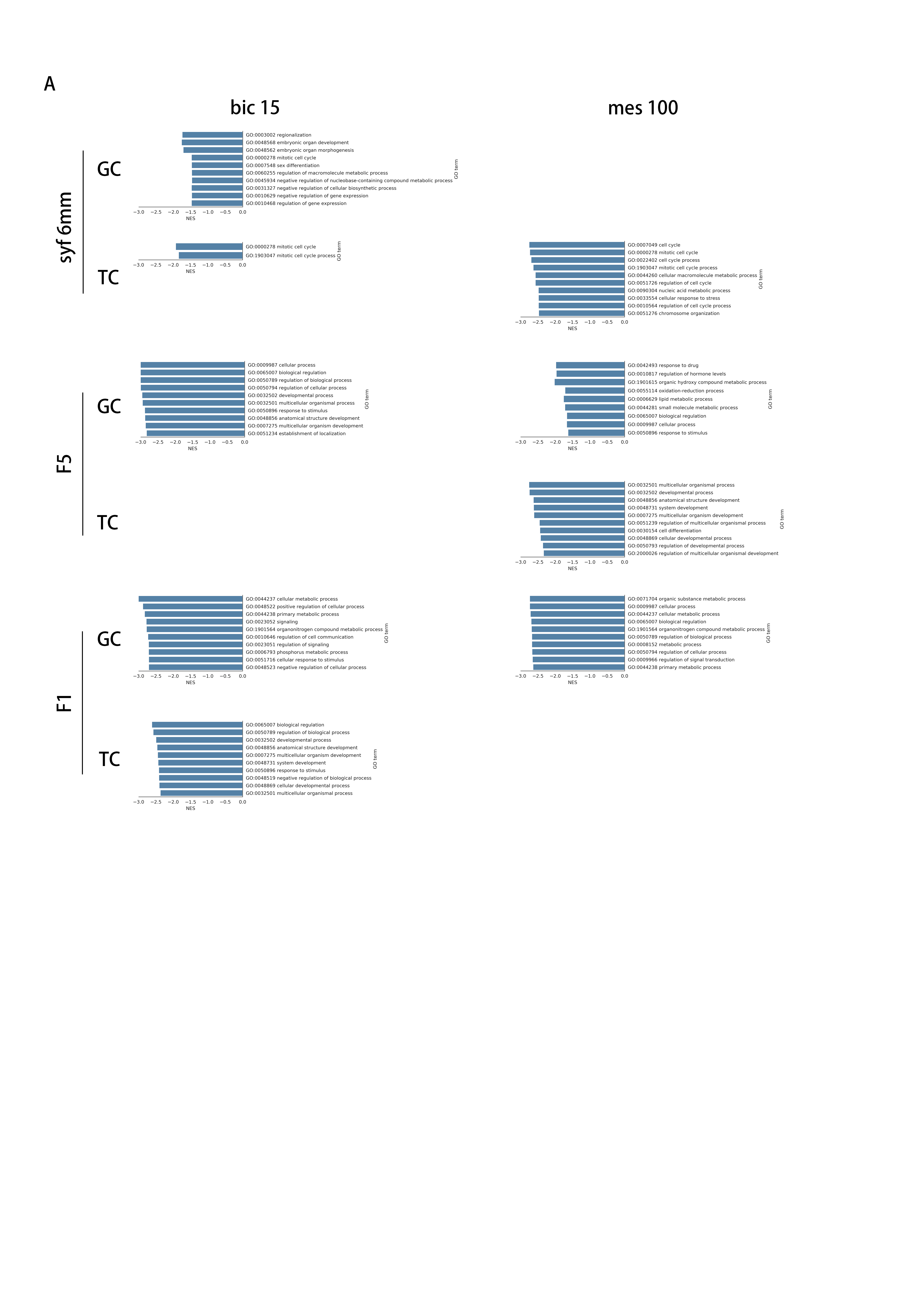

Supplement: Supplementary file 11 [file Image8.PNG]

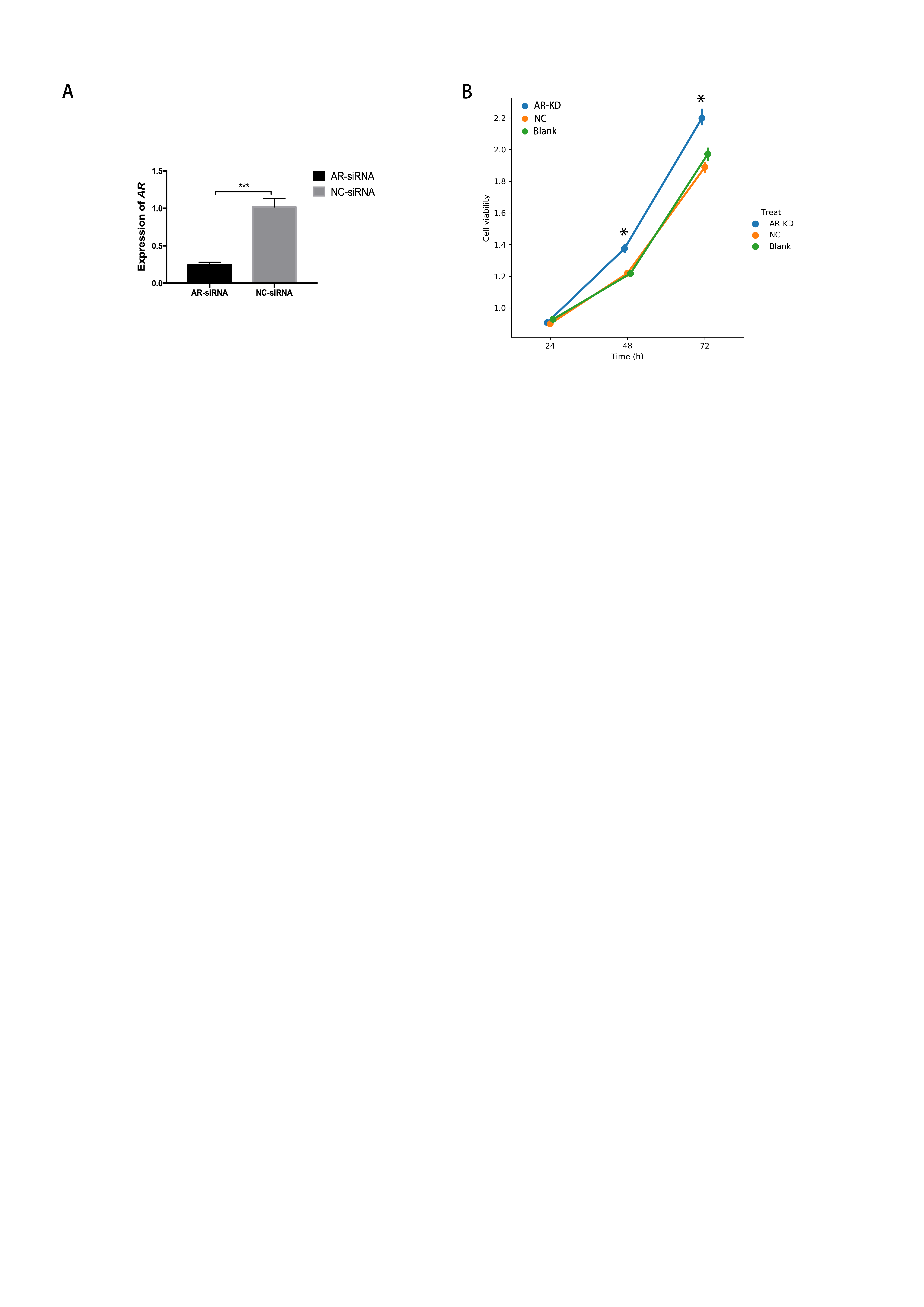

Supplement: Supplementary file 12 [file Image9.PNG]

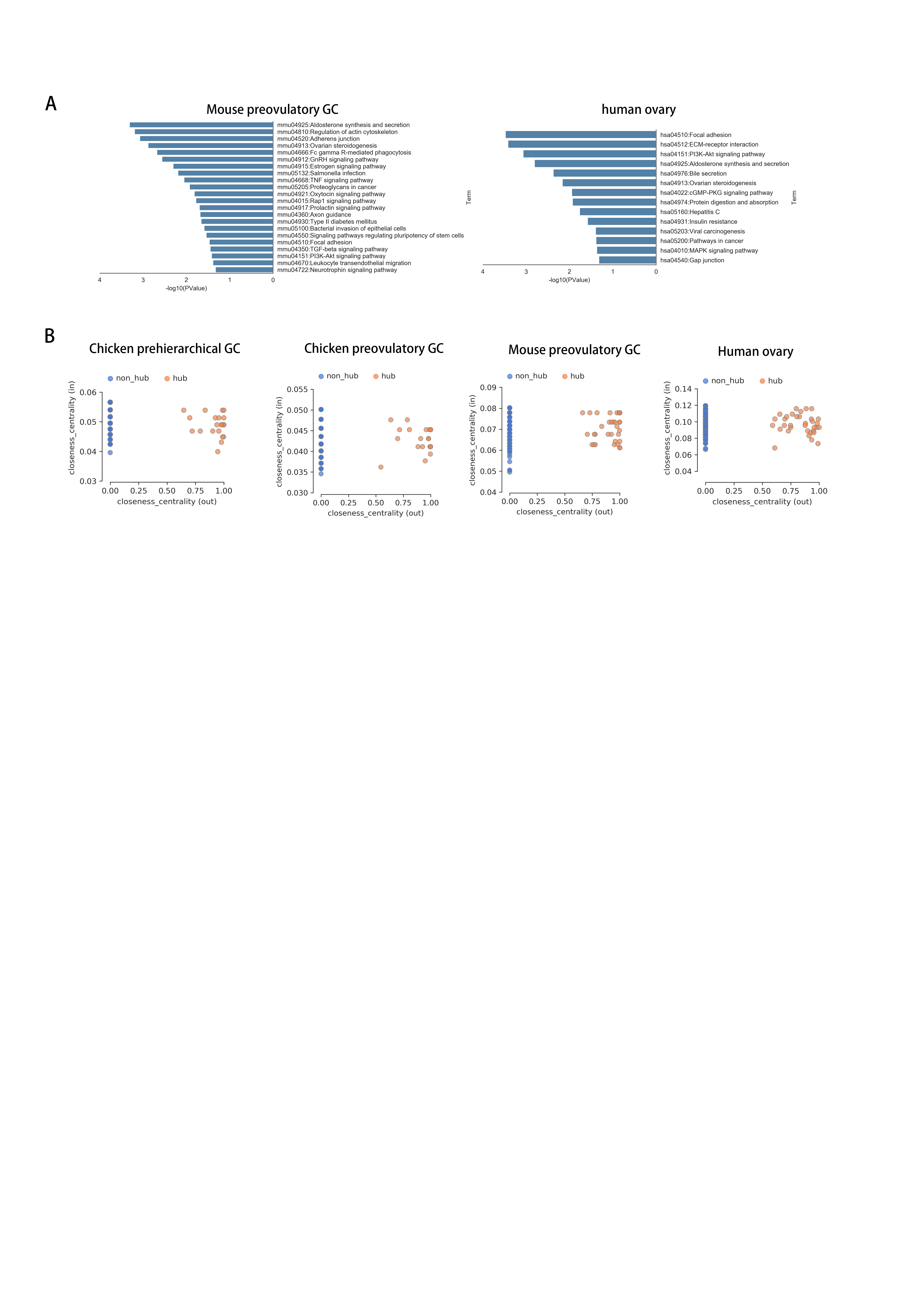

Supplement: Supplementary file 14 [file Image6.PNG]

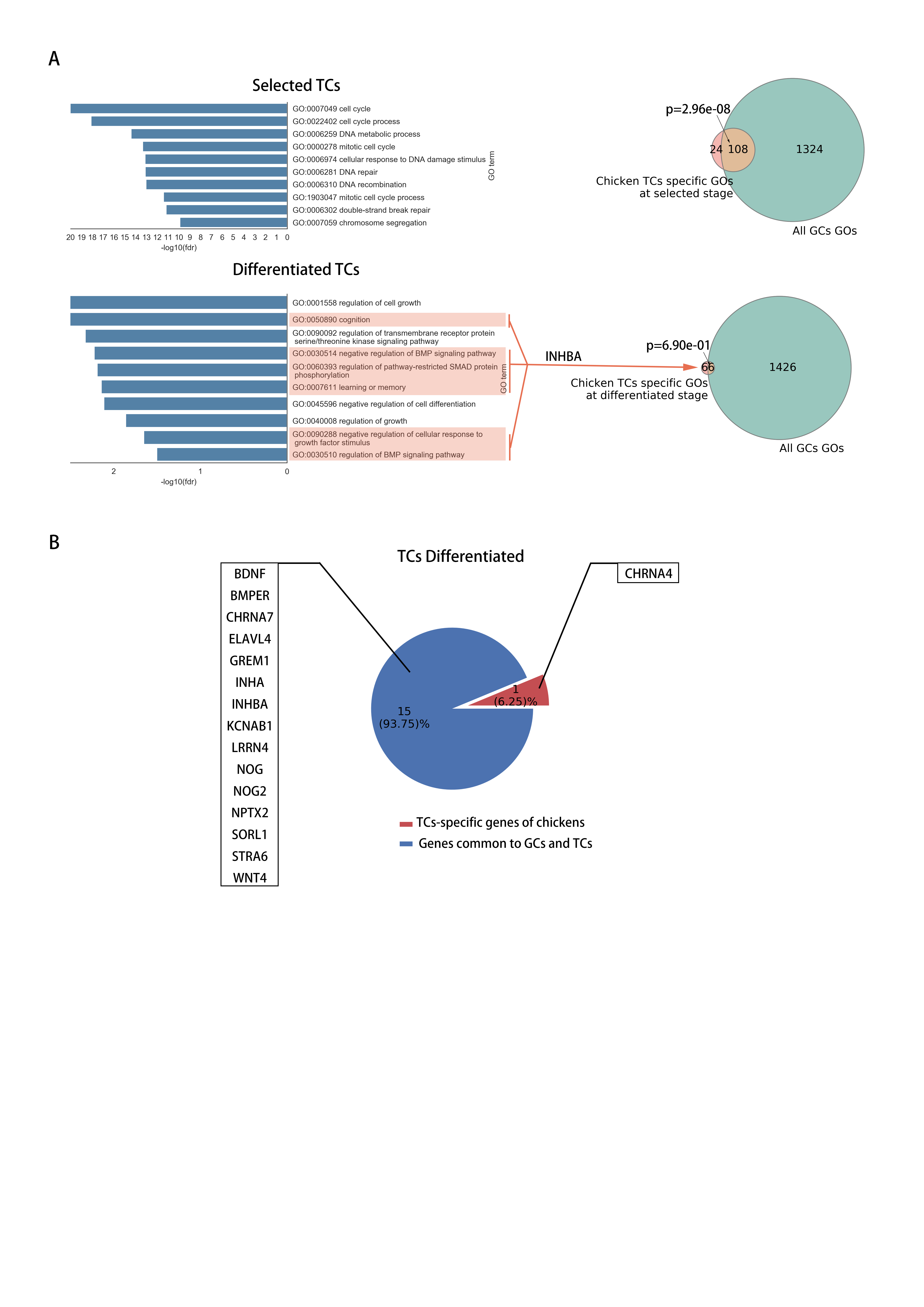

Supplement: Supplementary file 17 [file Image3.PNG]
