# Supplementary material for: Comparative Analysis Among Different Species Reveals That the Androgen Receptor Regulates Chicken Follicle Selection Through Species-Specific Genes Related to Follicle Development
Source: Front Genet. 2022 Jan 3;12:752976. doi: 10.3389/fgene.2021.752976 (PMC8762282; doi:10.3389/fgene.2021.752976)
Supplement: Supplementary file 18 [file DataSheet1.docx]

**Supplementary Information for**

Comparative analysis among different species reveals that the androgen receptor regulates chicken follicle selection through species-specific genes related to follicle development

Running title: Effect of AR on Chicken Follicular Development.

Ying Huang^a^, Wei Luo^a,b^, Xuliang Luo^a^, Xiaohui Wu^a^, Jinqiu Li^a,c^, Yan Sun^a^, Shuixin Tang^a^, Jianhua Cao^a^, and Yanzhang Gong^a, *^

*^a^Key Laboratory of Agricultural Animal Genetics, Breeding and Reproduction (Huazhong Agricultural University), Ministry of Education, Wuhan 430070, China;*

*^b^Guilin Medical University, Guilin, Guangxi, China;*

*^c^Central Laboratory, Affiliated Hospital of Putian University, Putian 351100, China.*

* Corresponding author. Email: poultry@mail.hzau.edu.cn (Y. Gong)

Methods

**RNA-Seq Data Processing**

The RNA-Seq raw data (FASTQ) were processed according to the standard procedure below unless otherwise stated. Cutadapt (v1.14) (1) was used to remove adapters and low-quality sequences from all raw data. The obtained clean reads were aligned to the reference genome using HISAT2 (v2.1.0) (2) with default parameters. Samtools (v1.7) (3) rmdup was used to remove the redundant sequences after alignment and Stringtie (v1.3.4d) (4) was used to calculate gene expression (reads count and FPKM). Human reference genome: GRCh38 (NCBI). Mouse reference genome: GRCm38 (Ensembl). Bovine reference genome: ARS-UCD1.2 (NCBI). Chicken reference genome: Gallus_gallus-5.0 (Ensembl).

In the correspondence of follicular developmental stages among species, we downloaded from public databases: (i) RNA-Seq raw data of GCs and TCs at the follicular development of bovine selection, differentiation, and preovulation (GSE34317) (5). (ii) scRNA-Seq gene expression (FPKM) data for human primordial, primary, secondary, antral, and preovulatory follicular developmental stages of GCs (GSE107746) (6). (iii) scRNA-Seq gene expression (reads count) data (GSE118127) for human pre-selected and selected follicular developmental stages of cumulus granulosa cells (CGCs), mural granulosa cells (MGCs), and theca cells (7). The data were converted into FPKM using an in-house script.

For the comparison of differential genes among stages of follicular development, to obtain the gene expression (reads count) of human preovulatory follicular GCs, we downloaded their scRNA-Seq raw data (GSE107746) and calculated the gene expression according to standard procedures.

The RNA-Seq data of small yellow follicles with high and low FSHR was download from GEO with access number GSE100673 (8).

**Correspondence of follicular developmental stages among species**

Using the gene expression data (FPKM) of GCs and TCs at each follicle development stage among species (human, bovine, chicken), the homologous gene expression matrix was constructed based on the NCBI HomoloGene database (https://ftp.ncbi.nih.gov/pub/HomoloGene/current/). Interspecies genes with the same name were also considered as homologous genes. Homologous genes expressed in at least one sample (FPKM ≥ 1) were retained. The Combat function of the sva (v3.30.1) R package (9) was used to remove the batch effect from different sample sources in the homologous gene expression matrix. The mean value of homologous gene expression for all samples at each follicular developmental stage was used to represent the homologous gene expression at that stage. Logarithmic scaling and z-score normalization of homologous gene expression by each stage of follicular development in each species. Finally, based on the normalized homologous gene expression data, hierarchical clustering (ward method) was performed for each stage of follicular development in each species.

The above method is also used to correspond to the stage of follicle development in human, rat, and chicken polycystic follicle syndrome.

To correspond more precisely to the developmental stages before and after follicular selection, we performed a dynamic time warping (DTW) analysis using the R package dtw (v1.20.1) (10) according to a previous study (11). The input distance matrix is based on the logarithmically scaled FPKM expression matrix computed with the dist function (method='dtw') of the R package proxy (v0.4.22). The parameters of the R package dtw are set to: keep = TRUE.

**Identification of Differentially Expressed Genes and GO Analysis**

In the comparison of follicle development-related genes across species, the gene differential expression was calculated based on reads count using DESeq2 (v1.22.2) (12). Genes with FPKM≥1, abs(logFC)≥1.5, and padj≤0.05 were defined as differentially expressed genes.

In the identification of genes related to follicular developmental stages in each species, genes differentially highly expressed at the respective follicular developmental stage compared to the selection stage were defined as related differentially expressed genes at the corresponding stage, and genes differentially highly expressed at the selection stage compared to the pre-ovulatory stage were defined as related differentially expressed genes at the selection stage (Table 1). Subsequently, Panther (http://pantherdb.org/) was used to enrich for the biological processes (GO biological process complete) and A-DaGO-Fun (v15.1) (13) was used to calculate GO semantic similarity. Finally, hierarchical clustering was done. In the hierarchical clustering tree structure, branches with distances less than 6 from each other were divided into clusters, resulting in a total of 42 clusters of semantically similar GO terms. After manual proofreading and labeling of the names, 35 clusters of biological processes were obtained.

**ChIP-Seq Data Processing**

ChIP-Seq raw data (FASTQ) was processed according to the following standard procedure unless otherwise stated. Cutadapt (v1.14) was used to remove adapters and low-quality sequences from raw data, and clean reads were aligned to the reference genome using Bowtie2 (v2.3.4.1) (14) with default parameters. Samtools (v1.7) rmdup was used to remove redundant sequences from the BAM files after alignment for subsequent analysis. We downloaded ChIP-Seq data of H3K27ac of mouse follicular granulosa cells (GSE115820) and human ovarian tissues (ENCFF052RXC, ENCFF282TNP) from public databases (15, 16). The human ovarian ChIP-Seq data are BAM files.

**Identification of Super-enhancers and Analysis of Their Associated Regulatory Networks**

MACS2 (v2.1.1.20160309) (17) was used for peak calling with parameters -B -p 1e-9, where chicken genome size is set to 1.2e9. Subsequently, the super-enhancers were identified using ROSE (v0.1) (18) with the default parameters, and the core transcriptional regulatory circuits (CRCs) were identified with CRCmapper (v1.0) (19). In the use of CRCmapper, genes with FPKM ≥ 1 were considered to be expressed genes and the chicken transcription factor database is derived from AnimalTFDB (v3.0) (20). All candidate CRCs were retained.

According to the previously described method (19), we constructed a transcriptional regulatory network based on super-enhancers. Super-enhancers, super-enhancer-associated genes, and their encoding proteins (transcription factors or non-transcription factors) were together considered to be a node in the transcriptional regulatory network. The node-to-node regulation depended on the prediction of transcription factor binding to super-enhancers. The closeness centrality (in), closeness centrality (out), and edge betweenness centrality of the transcriptional regulatory network were calculated using the closeness_centrality and edge_betweenness_centrality functions of networkx (v2.5), respectively.

**GSEA Analysis**

We used the genes included in the biological processes related to follicle development or super-enhancer associated genes as gene sets, and the gene expression data of GCs and TCs of corresponding species and follicle development stages as expression matrices (FPKM). The gene enrichment analysis was performed on GSEA (v3.0) software (21).

**Multiple Alignments Analysis**

All multiple alignments results were download from UCSC genome browser. The genomic regions homologous to the chicken AR super-enhancer or chicken poGCs super-enhancers across 77 species were extracted by in house scripts. The binding sites of candidate TFs screened by the chicken poGCs super-enhancers were calculated by fimo (v5.0.3) software (22). The gaps distribution was calculated by deeptools (v3.0.2) software (23).

**SI References**

1. Martin M. Cutadapt removes adapter sequences from high-throughput sequencing reads. EMBnetjournal. 2011;17(1):3.

2. Kim D, Paggi JM, Park C, Bennett C, Salzberg SL. Graph-based genome alignment and genotyping with HISAT2 and HISAT-genotype. Nat Biotechnol. 2019;37(8):907-15.

3. Li H, Handsaker B, Wysoker A, Fennell T, Ruan J, Homer N, et al. The Sequence Alignment/Map format and SAMtools. Bioinformatics. 2009;25(16):2078-9.

4. Pertea M, Kim D, Pertea GM, Leek JT, Salzberg SL. Transcript-level expression analysis of RNA-seq experiments with HISAT, StringTie and Ballgown. Nat Protoc. 2016;11(9):1650-67.

5. Walsh SW, Mehta JP, McGettigan PA, Browne JA, Forde N, Alibrahim RM, et al. Effect of the metabolic environment at key stages of follicle development in cattle: focus on steroid biosynthesis. Physiol Genomics. 2012;44(9):504-17.

6. Zhang Y, Yan Z, Qin Q, Nisenblat V, Chang HM, Yu Y, et al. Transcriptome Landscape of Human Folliculogenesis Reveals Oocyte and Granulosa Cell Interactions. Mol Cell. 2018;72(6):1021-34 e4.

7. Fan X, Bialecka M, Moustakas I, Lam E, Torrens-Juaneda V, Borggreven NV, et al. Single-cell reconstruction of follicular remodeling in the human adult ovary. Nat Commun. 2019;10(1):3164.

8. Wang Y, Chen Q, Liu Z, Guo X, Du Y, Yuan Z, et al. Transcriptome Analysis on Single Small Yellow Follicles Reveals That Wnt4 Is Involved in Chicken Follicle Selection. Frontiers in Endocrinology. 2017;8(317).

9. Johnson WE, Li C, Rabinovic A. Adjusting batch effects in microarray expression data using empirical Bayes methods. Biostatistics. 2007;8(1):118-27.

10. Giorgino T. Computing and Visualizing Dynamic Time Warping Alignments in R: The dtw Package. Journal of Statistical Software. 2009;31(7):24.

11. Cardoso-Moreira M, Halbert J, Valloton D, Velten B, Chen C, Shao Y, et al. Gene expression across mammalian organ development. Nature. 2019;571(7766):505-9.

12. Love MI, Huber W, Anders S. Moderated estimation of fold change and dispersion for RNA-seq data with DESeq2. Genome Biol. 2014;15(12):550.

13. Mazandu GK, Chimusa ER, Mbiyavanga M, Mulder NJ. A-DaGO-Fun: an adaptable Gene Ontology semantic similarity-based functional analysis tool. Bioinformatics. 2016;32(3):477-9.

14. Langmead B, Salzberg SL. Fast gapped-read alignment with Bowtie 2. Nat Methods. 2012;9(4):357-9.

15. Consortium EP. An integrated encyclopedia of DNA elements in the human genome. Nature. 2012;489(7414):57-74.

16. Davis CA, Hitz BC, Sloan CA, Chan ET, Davidson JM, Gabdank I, et al. The Encyclopedia of DNA elements (ENCODE): data portal update. Nucleic Acids Res. 2018;46(D1):D794-D801.

17. Zhang Y, Liu T, Meyer CA, Eeckhoute J, Johnson DS, Bernstein BE, et al. Model-based analysis of ChIP-Seq (MACS). Genome Biol. 2008;9(9):R137.

18. Whyte WA, Orlando DA, Hnisz D, Abraham BJ, Lin CY, Kagey MH, et al. Master transcription factors and mediator establish super-enhancers at key cell identity genes. Cell. 2013;153(2):307-19.

19. Saint-Andre V, Federation AJ, Lin CY, Abraham BJ, Reddy J, Lee TI, et al. Models of human core transcriptional regulatory circuitries. Genome Res. 2016;26(3):385-96.

20. Hu H, Miao YR, Jia LH, Yu QY, Zhang Q, Guo AY. AnimalTFDB 3.0: a comprehensive resource for annotation and prediction of animal transcription factors. Nucleic Acids Res. 2019;47(D1):D33-D8.

21. Subramanian A, Tamayo P, Mootha VK, Mukherjee S, Ebert BL, Gillette MA, et al. Gene set enrichment analysis: a knowledge-based approach for interpreting genome-wide expression profiles. Proc Natl Acad Sci U S A. 2005;102(43):15545-50.

22. Grant CE, Bailey TL, Noble WS. FIMO: scanning for occurrences of a given motif. Bioinformatics. 2011;27(7):1017-8.

23. Ramirez F, Ryan DP, Gruning B, Bhardwaj V, Kilpert F, Richter AS, et al. deepTools2: a next generation web server for deep-sequencing data analysis. Nucleic Acids Res. 2016;44(W1):W160-5.
